# Corn-Soybean Intercropping Improved the Nutritional Quality of Forage Cultivated on Podzols in Boreal Climate

**DOI:** 10.3390/plants10051015

**Published:** 2021-05-19

**Authors:** Muhammad Zaeem, Muhammad Nadeem, Thu Huong Pham, Waqar Ashiq, Waqas Ali, Syed Shah Mohioudin Gillani, Eric Moise, Sathya Elavarthi, Vanessa Kavanagh, Mumtaz Cheema, Lakshman Galagedara, Raymond Thomas

**Affiliations:** 1 School of Science and the Environment, Grenfell Campus, Memorial University of Newfoundland, Corner Brook, NL A2H 5G4, Canada; tpham@grenfell.mun.ca (T.H.P.); washiq@uoguelph.ca (W.A.); wali@grenfell.mun.ca (W.A.); ssmgmohioudi@grenfell.mun.ca (S.S.M.G.); mcheema@grenfell.mun.ca (M.C.); lgalagedara@grenfell.mun.ca (L.G.); 2 Department of Fisheries, Forestry and Agriculture, Government of Newfoundland and Labrador, St. John’s, NL A2H 7E1, Canada; 3 School of Environmental Sciences, University of Guelph, Guelph, ON N1G 2W1, Canada; 4 Atlantic Forestry Centre, 26 University Drive, Corner Brook, NL A2H 6J3, Canada; eric.moise@canada.ca; 5 Department of Agriculture and Natural Resources Delaware State University, 1200 N Dupont Hwy, Dover, DE 19901, USA; selavarthi@desu.edu; 6 Department of Fisheries, Land Resources, Government of Newfoundland and Labrador, St. John’s, NL A0L 1K0, Canada; VanessaKavanagh@gov.nl.ca

**Keywords:** forage quality, forage fatty acid composition, forage mineral composition, silage corn, forage soybean

## Abstract

Intercropping systems could be a potential source of nutrient-rich forage production in cool climates on podzolic soils common in boreal ecosystems. In this study, we evaluated the effects of corn–soybean intercropping (IC) on the nutritional quality of forage. Two silage corn varieties were cultivated as monocropping (MC) or were intercropped with three forage soybean varieties using a randomized complete block design. IC significantly increased the crude protein (22%) and decreased the acid detergent (14%) and neutral detergent (6%) fibers. Forage net energy, total digestible nutrients, ash, dry matter intake, digestible dry matter and relative feed value were also significantly increased (*p* ≤ 0.05) in the IC treatments compared to corn MC. The macro and micro nutrients were higher in IC than corn MC. Intercropping increased the omega 3 fatty acid (FA) contents (67%) compared to corn MC. IC also increased the active microbial community in the plant root zone, which may contribute to the improvement in forage nutritional quality because the active soil microbial community composition showed significant correlations with soluble sugars, soluble proteins and potassium contents of the forage. These results demonstrate that corn–soybean IC could be a suitable cropping system to increase the nutritional quality of forage cultivated on podzols in boreal climates. The resultant forage has the potential to be a source of high-value animal feed for livestock production in cool climate regions of the world.

## 1. Introduction

Corn (*Zea mays* L.) is the third most important cereal crop, after rice and wheat, cultivated for human and animal consumption. As a source of animal feed, silage corn has the ability to provide energy-rich forage with relatively high nutritional value, especially for the dairy industry [1,2,3]. Corn is grown across all the crop production zones globally and is one of the most versatile crops because of its adaptability under various climatic conditions, including boreal climates [4]. Silage corn is enriched with starch, water soluble carbohydrates (WSC) and fiber [5,6] and can be safely fed to animals at all growth stages. Even though silage corn is an excellent source of high-energy feed [4], the forage produced is low in protein [7,8,9]. Due to the low protein content in corn-based forages, additional supplements must be added to balance the diets of both dairy and meat producing animals. Protein is an important nutrient required for animal growth, reproduction and maintenance [10]. The use of legumes intercropped with corn or other forage species from the *Poaceae* (cereal or grass) family have been proposed as a suitable alternative to enhance the nutrient composition of forage produced for animal feed. This is particularly important for livestock production in boreal climates where protein supplementation is a high input cost [1,11,12,13]. Legumes are rich in protein, essential fatty acids, macro and micro minerals, and ash compared to grasses/cereals [1,11,12,13].

Soybean (*Glycine max*) is a fast growing, protein-rich legume that can be intercropped with corn to improve the protein and mineral contents in the forage [14]. Currently, two-thirds of the world’s protein concentrates used for feed in the livestock industry is obtained from soybean [15]. One negative factor associated with legume-based forage is that the dry matter content is low [16,17]. Consequently, corn intercropped with legumes could be a good approach to increase overall feed quality compared to forage obtained from monocropped corn or soybeans [14,18]. Intercropping (IC) is defined as the growing of two or more crops during the growing season, simultaneously in the same field [19,20,21]. In fact, the positive effects of corn–soybean IC on forage production and quality have been documented in several studies [14,22,23,24,25,26,27]. However, there is very limited work related to the influences of corn–soybean IC on the nutritional quality of forage cultivated on podzols under cool climatic conditions in boreal regions. Podzols are the predominant soils found in boreal environments and are characterized by high quartz content. These soils tend to be sandy, acidic and well drained; they contain bleached eluvial and alluvial horizons with accumulated humus, metal oxides (Al and Fe) and primary minerals [28]. Agricultural production on podzols is increasing, as the growth in global population leads to more people living in boreal regions. As such, there is a growing need to cultivate more food to feed the livestock and human populations in these regions. Though podzols are not ideal for agriculture, their predominance in the boreal landscape necessitates their use for agricultural production. It is currently unknown how corn–soybean IC influences the forage nutritional quality when cultivated on podzols in boreal climates or whether the active microbial populations in the IC corn and soybean have any relationship with the forage nutritional quality produced.

The present study was conducted in Newfoundland, Canada, and is representative of northern or boreal climates [29]. It addresses the paucity of information available on how silage corn intercropped with upright (US) or vine type (VS) forage soybean affects the nutritional quality (net energies, relative feed value (RFV), fatty acid composition (omega 3 and omega 6) and macro and micro mineral of the forage produced. Furthermore, very little is known about whether there are any relationships between the active soil microbial composition and forage nutritional quality when cultivation takes place on podzols in cool climate forage production systems. The soil microbial community can enhance the availability of nutrients for crop growth [30]. Most of the nutrients in the soil are present in bound forms, and they can be mineralized into plant useable form by soil microbes such as bacteria, protozoans and fungi [31,32]. This nutrient mineralization may lead towards high nutrient uptake and improvement in the nutritional composition of the forage crop produced. Forages are the main source of essential polyunsaturated fatty acids (PUFAs), for example, linoleic (C18:2n-6) and α-linolenic acids (C18:3n-3) in animal feed, and they have the ability to transform the milk or meat fatty acid (FA) composition [33,34]. In addition, both linoleic and linolenic FAs are of prime significance in producing beef with enhanced levels of omega 3 and conjugated linoleic acids (CLAs) [35]. CLAs and omega 3 fatty acids play significant roles in reducing cardiovascular disease, cancer, diabetes and obesity in humans [36].

We hypothesized that corn–soybean IC will increase the nutritional quality of forage cultivated under cool climatic conditions on podzolic soils via enhancement of the protein, essential FAs and mineral contents as well as a decline in the fiber contents compared to forage cultivated as monocultures. We also hypothesized that the soil active microbial composition may play a role in improving the overall forage quality. Therefore, this study aimed to investigate the effects of VS or US intercropped with silage corn on the nutritional quality (proteins, fibers, energy, minerals and fatty acid contents) and to seek the relationships between the active microbial community structure and the nutritional quality of the forage produced.

## 2. Materials and Methods

A two-year field-based study was conducted at Pynn’s Brook Agriculture Research Station, Pasadena (49.0936° N, 57.5359° W), Newfoundland, Canada. The soil at the research station has been assessed as podzols, and the station has been in use for research trials for over 20 years. In this study, two silage corn genotypes (Yukon-R: Brett Young^TM^, Winnipeg, MB, Canada; DKC26-28RIB: DEKALB^®^, DeKalb, Illinois, USA) were selected based on their agronomic performance [37,38,39] and were either monocropped or intercropped with three soybean genotypes (S1: Big Fellow RR, S2: Game Keeper RR, S3: Kester’s Bob White). Kester’s Bob White (S3) is a trailing (vine type) soybean variety obtained from the Department of Agriculture, USA, whereas S1 and S2 are upright varieties sourced from Delaware State University, USA. The forage soybean varieties were selected because of their high biomass production capabilities (upright varieties can grow up to 10 ft); Kester’s Bob White is a trailing (vine type) variety that can reach up to 20 ft. None of these crop varieties and combinations have ever been assessed in boreal climates or on podzols prior to our work [40]. The crop was sown on 20 June and 30 May in 2016 and 2017 [40]. The experimental design was a fully replicated randomized complete block design (RCBD), with a total of 11 treatments consisting of three replications per treatment. The dimension of each experimental treatment plot was 5 m × 6 m. Further details about seeding rate, seed inoculation, fertilization applications, weed control and crop harvesting can be found in our previous work [40]. The soil chemical properties and weather data during both growing seasons are given in Table 1 and Table 2.

### 2.1. Corn–Soybean Forage Quality Analysis

Three plants were selected randomly within each replication at harvesting and cut into small pieces. The pieces obtained from the three plants from each treatment replicate were pooled to make a composite sample (i.e., three plants were used per replicate, and three replicates were used for each of the 11 treatments). The samples were then dried in an oven at 60 °C (ShelLab FX14-2, Sheldon Manufacturing Inc., Cornelius, NC, USA) until constant plant dry weight was achieved. The samples were then ground using a CryoMill (Retsch^®^ GmbH, Haan, Germany), and the powdered samples were sent for forage quality analysis at Activation Laboratories Ltd. (Ancaster, ON, Canada), a member laboratory of the Dairy One Feed and Forage Analyses Laboratories (Ithaca, New York, USA). Near Infrared Reflectance Spectroscopy (Foss NIR System Model 6500 Win ISI II v1.5, Hillerod, Denmark) was used to determine the forage nutritional quality, including forage proteins (crude protein (CP), available protein (AP), soluble protein (SP)), fibers (acid detergent fiber (ADF), neutral detergent fiber (NDF)), TDN (total digestible nutrient), ash contents, simple sugars (SS), water soluble carbohydrates (WSC), forage energy (net energy of lactation (NEL), net energy of maintenance (NEM), net energy gain (NEG)), DDM (digestible dry matter), DMI (dry matter intake) and RFV (relative feed value) according to reference models published in the literature for animal feed composition and analysis [41,42,43].

### 2.2. Determination of Forage Mineral Nutrients

The 250 mg dried and sieved (450 um) forage sample was weighed into pre-cleaned digestion vessels. Aliquots (10 mL) of concentrated (67–70%) trace metal grade nitric acid (HNO_3_) (Catalog No. A509P212, Fisher Scientific, Ottawa, ON, Canada) was added to each sample vessel. All digestion vessels were tightly capped, and samples digested using a microwave digestion system (Multiwave Go Microwave Digestion System; Anton Paar, USA) for 20 min to ensure complete sample digestion. The microwave digestion process was comprised of two steps, as given in Table 3. After sample digestion was completed, the vessels were cooled to room temperature and carefully opened in the fume hood, and the samples were filtered into 50 mL Nalgene plastic bottles, then stored at ±4 °C. The samples were later diluted at 1:100 and 1:1000 ratios for determining mineral nutrient composition using an Inductively Coupled Plasma Mass Spectrometer (Thermo Scientific iCAP Q- ICP-MS, Brampton, ON, Canada). The instrument operation conditions were as follows: auxiliary gas flow 0.79 (L min^−1^), nebulizer gas flow 1.01 (L min^−1^), plasma gas flow 14 (L min^−1^), RF power 1548 (W), and dwell time 0.01 (s). A high purity multi element (43 elements) ICP‒MS standard solution (IV‒ICPMS‒71A; Inorganic™ Ventures, Inc., Christiansburg, VA, USA) was used for external calibration (0, 2, 5, 10, 20, 50, 100, 200, 300, 500 parts per billion) with linear regression values (R^2^) for each element ranging from 0.971–1.00.

### 2.3. Forage Fatty Acid Extraction

Powdered forage samples were extracted by the Folch [44] method, with some modifications. In brief, 250 mg of the ground, dried plant samples were transferred to glass centrifuge tubes containing 2.5 mL of (2:1: 0.0003; *v*/*v*/*wt*.) chloroform: methanol and butylated hydroxytoluene. The supernatant was collected following centrifugation at 5000 rpm for 15 min. Potassium chloride (1 mL of 0.25%) was next added to the supernatant, and the sample mixture was incubated in an oven for 10 min at 70 °C. After incubation, the organic layer (bottom layer) was transferred using glass Pasteur pipettes into pre-weighed 4 mL glass sample vials. The samples were then dried under N_2_, and the vials were re-weighed to determine the amount of lipids recovered [44,45]. The recovered samples were then re-suspended in 2 mL chloroform: methanol (2:1 *v*/*v*), stoppered with PTFE (Polytetrafluoroethylene) lined caps and used for FA methyl ester (FAME) analysis.

### 2.4. Fatty Acid Methyl Esters (FAMEs) Analysis

Aliquots of the extracted lipids (200 µL) were transferred to 2 mL sample vials, and 50 µL of C19:0 fatty acid (1mg/mL dissolved in 2:1 chloroform: methanol) was added as an internal standard. The samples were then dried under N_2_, and 200 µL methanolic HCl-3N (Sigma-Aldrich, ON, Canada) was added to the samples. The mixture was vortexed and then incubated at 90 °C for 30 min. After incubation, 0.8 mL distilled water was added to each sample, and the FAMEs were extracted three times using 500 µL hexane each time. The hexane fractions were pooled, dried under N_2_, then re-suspended into 100 µL hexane before being transferred, using Pasteur pipettes, into GC vials fitted with inserts (200 uL). Aliquots of the sample were injected in splitless mode into a gas chromatography-flame ionization detection (GC-FID) machine (Trace 3000, Thermo Scientific, Brampton, ON, Canada). The FAME peaks were identified through comparison of retention times obtained from commercial standards (Supelco 37 Component FAME Mix; Sigma Aldrich, ON, Canada). Quantification of individual FA was done using standard curves prepared from the standard mixture and values presented as g/kg of forage dry matter (DM).

### 2.5. Microbial Fatty Acid Analysis

Phospholipid fatty acid (PLFA) analysis was used to determine the active microbial biomass present in the soil, as described in details in Zaeem et al. (2019). In brief, 4 g rhizosphere soil samples that were removed from the uprooted plant roots were extracted for PLFA using a modified Folch method [44]. PLFAs in the samples were analyzed with gas chromatography-mass spectrometry (GC-MS) and gas chromatography-flame ionization detection (GC-FID). The identified PLFAs were used as markers to assess the active microbial communities in the soil samples when the forage reached maturity and was harvested for nutritional analysis [44].

### 2.6. Statistical Analyses

The trends in the data were similar between years; thus, the data was pooled over the two growing seasons. All statistical analyses were conducted using XLSTAT (Premium Version, 2019 Addinsoft, Long Island, NY, USA) and Statistix-10 software programs (Analytical Software, Tallahassee FL, USA). All measurements of biochemical parameters (PLFAs, minerals, fatty acids, etc.) were done in quadruplicate. One way analysis of variance (ANOVA) was used to assess the effects of different treatments on forage nutritional quality parameters. Fisher’s Least Significant Difference (LSD) was used to compare the means when the treatment effects were significant at α = 0.05. Principal component analysis (PCA) and Pearson’s correlation coefficients (r) were used to test the relationships between treatments, active microbial community composition, and nutrients present in the forage. Graphs generated from the data were created using Sigma Plot 13.0 (Systat Software Inc., San Jose, CA, USA).

## 3. Results

### 3.1. Forage Nutritional Quality

We observed significant improvement in the overall nutritional quality of corn–soybean forage produced in the IC treatments compared to when cultivated as monocultures (Table 4). The corn–soybean IC treatments resulted in significantly higher CP and AP than corn MC treatments (Table 4). The SP contents were significantly higher in the IC treatments compared to either corn or soybean cultivated as MC. The highest SP was observed in the S3C1 (47.8%) treatments, while the lowest was recorded in the S1 (35.7%) treatments.

The highest CP was observed in S1 (19.4%), and the lowest in C2 (10.3%) (Table 4). Similar to the protein content, IC of silage corn with forage soybean had significant effects on the forage fiber content (ADF and NDF) when cultivated on podzols in cool climatic conditions (Table 4). We observed that C1 and C2 treatments produced the highest ADF contents (37.7% and 36.8%, respectively) compared to all other treatments; the lowest ADF was observed in the S2C2 IC treatments (29.9%). Conversely, soybeans cultivated as monocrops produced forage with significantly lower NDF content (Table 4). The highest NDF content was observed in the corn MC treatments (C1 57.7), while forage produced in the IC treatments had an NDF content between that of corn and soybean MC treatments. The fiber content was similar in forage obtained from both vine and upright soybeans, regardless of whether they were cultivated as monocrops or intercropped with silage corn over the duration of the study (Table 4).

As expected, the WSC was highest in forage produced in the corn MC treatments. As such, all the IC treatments produced forage with significantly higher WSC contents than soybean MC treatments. When soybean was grown as a monocrop, higher levels of WSC were present in the forage obtained from upright soybean varieties than from the vine-type variety. The WSC level was highest in C2 (17.3%), and lowest in S3 (5.35%). Conversely, the SS concentration was significantly higher in IC compared to corn and soybean MC treatments during the growing seasons. The highest SS content was measured in S3C1 (11.4%), while the lowest value was recorded in S2 (5.73%).

The overall ash concentration was significantly higher in soybean cultivated as MC compared to all other treatments. The vine-type variety (S3) produced forage with significantly higher ash content compared to the upright varieties (S1, S2) cultivated as monocrops. The lowest ash content was observed in the corn MC treatments, while the IC treatments were intermediate, between the soybean and corn MC treatments (Table 4).

Overall forage produced in the IC treatments had significantly higher TDN, NEL, NEM and NEG compared to the MC treatments (Table 5). The upright varieties produced forage with higher TDN, NEL, NEM and NEG values than vine soybeans cultivated as MC. The highest NEL was recorded in S1 and S2 (1.30 Mcal/kg), while the lowest was recorded in S3 (1.08 Mcal/kg) during the growing seasons. Soybean MC produced forage with the highest estimated DMI, followed by the IC and the corn MC treatments. The overall DDM, on the other hand, was significantly higher in the IC treatments. As such, the highest DDM was recorded in S2C2 (65.6%), and the lowest was recorded in C1 (59.5%) over the growing seasons (Table 5).

As expected, the RFV was higher for soybean cultivated as MC than both IC and corn MC treatments (Table 5). The trend was similar for RFV as was observed for DMI (soybean MC > corn–soybean IC > corn IC). The highest values were recorded for S2 (144%), and the lowest were recorded in C1 (95%). All the IC treatments were higher in RFV compared with the corresponding corn MC treatments.

### 3.2. Mineral Composition of Corn and Soybean Forage Obtained Following Cultivation as Mono and Intercrop

Forage obtained from soybeans cultivated as monocrops contained the highest macronutrient content, while the forage obtained from corn cultivated as a monocrop had the lowest macro nutrients. When corn and soybeans were cultivated as intercrops, the macronutrient level in the forage produced was intermediate, between that of the forage produced from corn and soybean MC treatments.

The highest bioaccumulation of Ca, K and Na was observed in S2 (1.60 g/kg, 17.5 g/kg, and 59.2 mg/kg, respectively), while the highest levels of Mg and P were observed in S1 (7.07 and 3.61 g/kg, respectively) (Table 6). Conversely, the lowest levels of Ca and P were recorded in C1 (0.14 g/kg and 2.10 g/kg, respectively), and the lowest levels of Mg, K and Na were in C2 (2.64 g/kg, 9.38 g/kg and 13.4 mg/kg, respectively).

Soybean cultivated as a monocrop generally produced forage with the highest micronutrient contents, followed by the IC treatments; corn MC produced forage with the lowest micronutrient contents. Surprisingly, when the upright and vine-type soybean varieties were intercropped with silage corn, the micronutrient content of the forage produced was similar between the IC treatments, regardless of the soybean variety that was intercropped (Table 7). The concentration ranges of the micro nutrients Zn, Fe, B, Mn, Cu and Co were 10.6–35.4 mg/kg, 247–1244 mg/kg, 5.22–32.1 mg/kg, 56.9–206 mg/kg, 7.27–16.8 mg/kg and 0.36–1.93 mg/kg, respectively (Table 7). Overall, the soybean MC produced forage with superior micronutrient content compared to the IC treatments. 

### 3.3. Fatty Acid Composition of Forage Obtained from Corn and Soybean Cultivated as Monocrops or Intercrops

Overall, the total FAs were higher in IC compared with corn MC treatments. Means of saturated fatty acids (SFAs) were higher in soybean MC than corn MC and IC treatments, while the monounsaturated fatty acids (MUFAs) were higher in IC than both corn and soybean MC. Briefly, the highest C16:0 (g/kg DM) was observed in S1 (2.30), while the lowest content was recorded in C1 (1.73) (Table 8). The maximum C18:1n-9 content (g/kg DM) was recorded in S1C2 (1.85), while the lowest was recorded in S2 (0.50). The C18:2n-6 (g/kg DM) ranged from 1.59 in S2 to 2.90 in C2. The highest C18:3n-3 (g/kg DM) was observed in S1 (1.84) and the lowest value in C1 (0.46) (Table 8). IC of corn with soybean increased the C18:3n-3 (g/kg DM) contents in the forage, while a decrease was observed in C18:2n-6 when compared to corn MC treatments.

**Figure 1:**
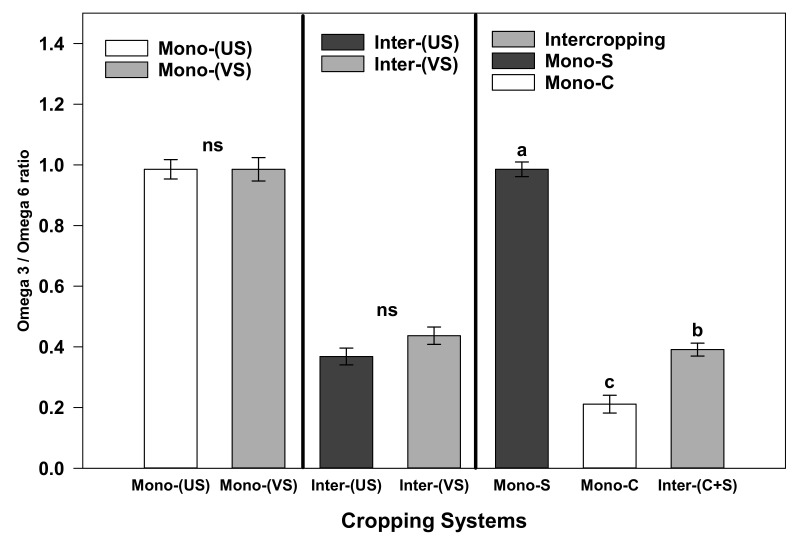
The ratio between omega 3 and omega 6 FAs under corn and soybean cropping systems during the 2016 and 2017 growing seasons. Explanations: ns = non-significant. The error bar represents SE. Different letters indicate differences between the cropping systems at LSD = 0.05. Mono C = Monocropping Corn; Mono S = Monocropping Soybean; Mono-(US) = Monocropping Upright Soybean; Mono-(VS) = Monocropping Vine Soybean; Inter-(US) = Upright Soybean Intercropped with Corn; Inter-(VS) = Vine Soybean Intercropped with Corn.

The highest total FAs (7.67 g/kg DM) were recorded in S1C2, while the lowest level (5.98 g/kg DM) was observed in C1(Table 8). Upright varieties contained higher omega 3 FA contents than the vine-type variety when cultivated as MC (Figure 1). However, the omega 3/omega 6 ratio was significantly higher in IC than corn MC treatments (Figure 1).

The PUFAs were the major contributor (43–53%) to the total FA profile of the forage. Saturated and MUFAs contributed (29–37%) and (14–26%), respectively, of the total FAs. Overall, SFAs were lower (29% to 37%) compared to unsaturated FAs (UFAs) (65% to 69%) during both years. In all the forage obtained from the monocropped or intercropped treatments, C16:0 and C18:2n-6 were the major FAs, with an average contribution of 29% and 33% of total FA measured, respectively. During the whole season, the FA composition was as follows: C18:2n-6 (33%) > C16:0 (29%) > C18:1n-9cis (17%) > C18-3n-3 (15%) > C18:0 (4%) > C16-1n-7 (3%). The contribution of the first four FAs (C18:2n-6, C16:0, C18:1n-9cis, C18-3n-3) was 98% of the total FAs in the forage. The rest of the FAs contributed only 2%.

### 3.4. Relationships between the Cropping Systems, Active Microbial Population and Quality of the Forage When Cultivated on Podzols in Cool Climates

The first axis (F1) of the RDA biplot explained 41.32%, and the second axis (F2) explained 31.11% of the total variation in the data. The first axis (F1) correlated positively with ash, CP, AP, DMI and RFV but was negatively correlated with SS, SP, WSC and NDF. The second axis (F2) was positively correlated with net forage energies, DDM and TDN, while a negative correlation was observed with ADF contents.

**Figure 2:**
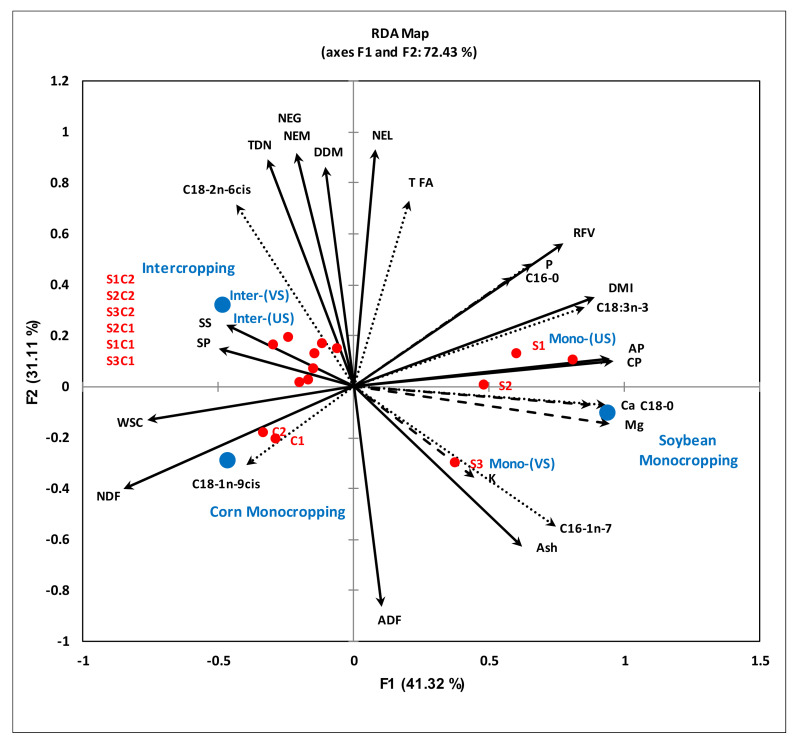
Redundancy analysis (RDA), showing the relationship between the fodder quality parameters and FA contents of forage obtained from corn and soybean cultivated as either monocrops or intercrops under cool climatic conditions. The FA contents are represented by dashed arrows, fodder quality by bold solid arrows and minerals by dotted arrows. CP (crude protein); AP (available protein); SP (soluble protein); SS (soluble sugars); TDN (total digestible nutrients); NEL (net energy lactation); NEM (net energy maintenance); NEG (net energy gain); WSC (water soluble carbohydrates); NDF (neutral detergent fiber); ADF (acid detergent fiber); DMI (dry matter intake); DDM (digestible dry matter); RFV (relative feed value); TFA (total fatty acids). Mono-(VS) = vine soybean monocropping; Mono-(US) = upright soybean monocropping; Inter-(US) = upright soybean intercropped with corn; Inter-(VS) = vine soybean intercropped with corn.

**Figure 3:**
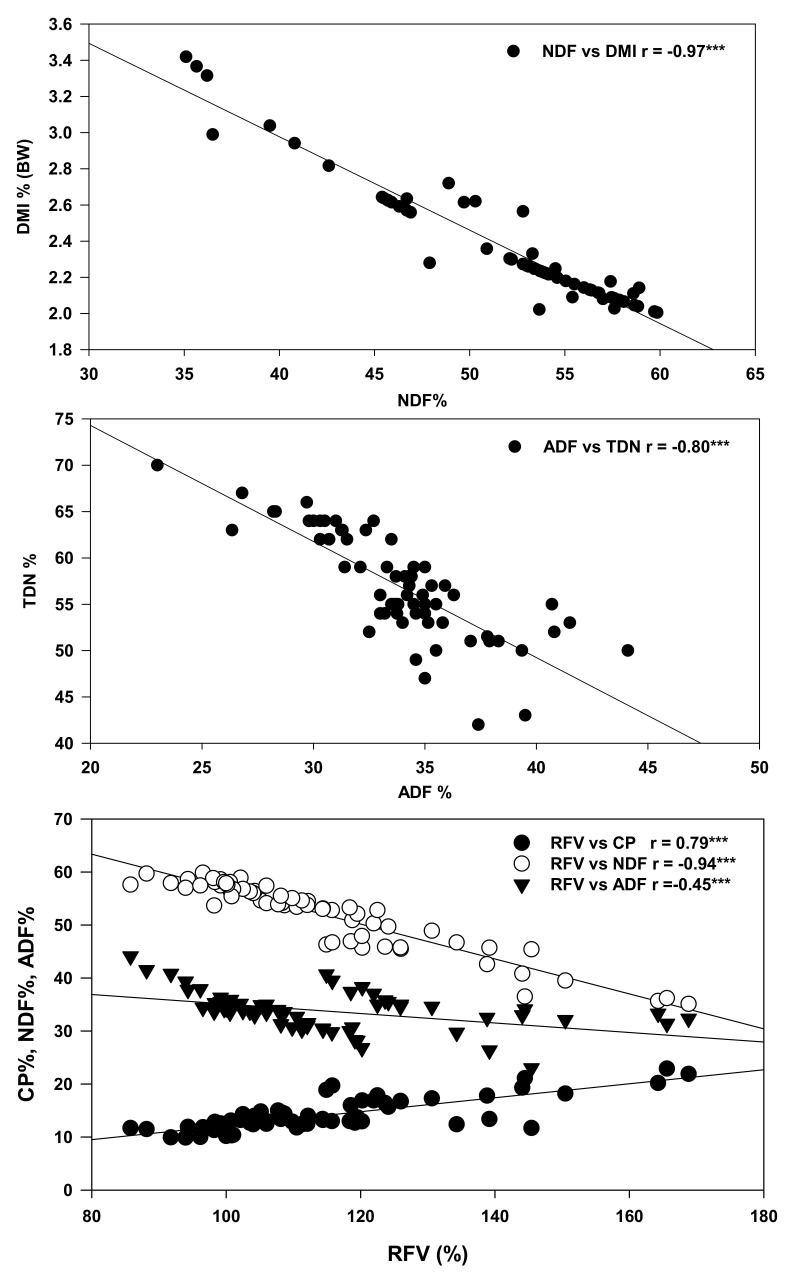
Pearson correlation between NDF and DMI; ADF and TDN; RFV and CP, ADF and NDF for different corn–soybean monocropping and intercropping treatments. CP = crude protein; NDF = neutral detergent fiber; ADF = acid detergent fiber; RFV = relative feed value; DMI = dry matter intake; TDN = total digestible nutrients; ns = non-significant; * correlation is significant (*p* ≤ 0.05); ** (*p* ≤ 0.01); *** (*p* ≤ 0.001), n = 66 for all parameters.

The corn and soybean MC and the IC treatments clustered in separate quadrants with specific quality parameters. For example, DDM, NEM, NEG, TDN, SS, SP and C18:2n-6 were the factors most influenced by IC treatments, and as such they clustered with the IC treatments in a separate quadrant of the biplot compared to corn and soybean MC treatments (Figure 2). A positive correlation was observed between the forage quality and the major FAs. Conversely, ADF, NDF, ash, WSC and SS showed a negative correlation with the major forage FAs. Further confirmation of these significant relationships was observed following Pearson’s correlation analysis (Table 9). For example, the C18:3n3 level was highly correlated with CP (r = 0.86), AP (r = 0.82), ADF (r =−0.86), DMI (r = 0.90) and RFV (r = 0.86). Conversely, the C18:2n-6 level was highly correlated with ADF (r = −0.55), ash (r = −0.81), TDN (r = 0.70), NEM (r = 0.69), NEG (r = 0.69), NEL (r = 0.61) and DDM (r = 0.55). Similar to C18:3n-3, the C18:0 level was highly correlated with the CP (r = 0.86), AP (r = 0.83), NDF (r = −0.71), DMI (r = 0.77) and RFV (r = 0.65). Additionally, several nutritional parameters were observed to be highly (significantly) correlated with the quality of the forage produced in the cropping systems when cultivated on podzolic soil under cool climatic conditions in boreal ecosystems. These include RFV, which was highly correlated with CP (r = 0.79), ADF (r = −0.45), NDF (r = -0.94); ADF with TDN (r = −0.80); NDF with DMI (r = −0.97) (Figure 3).

**Figure 4:**
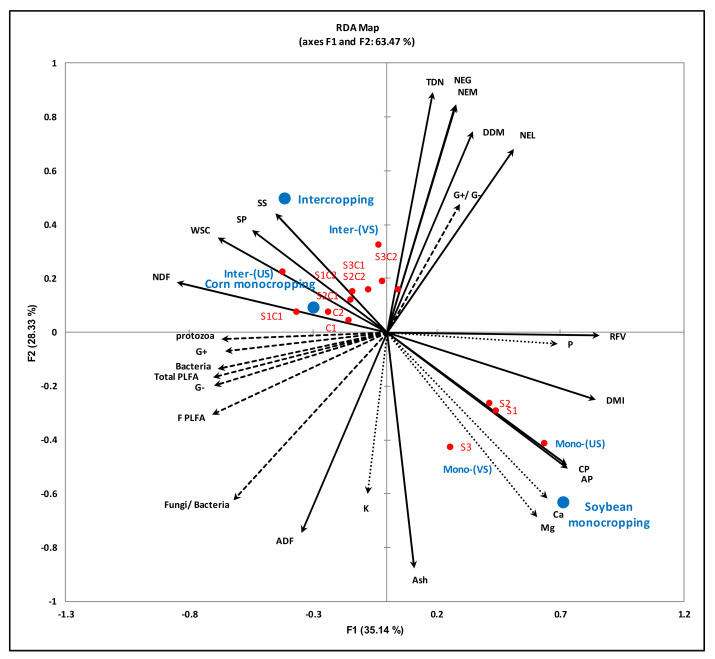
Redundancy analysis (RDA) showing the relationship between the fodder quality parameters and rhizosphere soil microbial community. The microbial community (PLFAs) are represented by dashed arrows, minerals by dotted arrows and fodder quality by solid arrows. G+ (gram positive bacteria); G- (gram negative bacteria); PLFAs (phospholipid fatty acids); CP (crude protein); AP (available protein); SP (soluble protein); SS (soluble sugars); TDN (total digestible nutrients); NEL (net energy lactation); NEM (net energy maintenance); NEG (net energy gain); WSC (water soluble carbohydrates); NDF (neutral detergent fiber); ADF (acid detergent fiber); DMI (dry matter intake); DDM (digestible dry matter); RFV (relative feed value); TFA (total fatty acids); Mono-(VS) = vine soybean monocropping; Mono-(US) = upright soybean monocropping; Inter-(US) = upright soybean intercropped with corn; Inter-(VS) = vine soybean intercropped with corn.

The RDA biplot (Figure 4) shows the relationship between the forage nutritional qualities and soil active microbial community. The first axis (F1) of the RDA biplot explained 35.14% and second axis (F2) 28.33% of the total variation in the data. Only the SP, SS and K were observed to be significantly correlated with the soil microbes present in the cropping systems evaluated.

**Figure 5:**
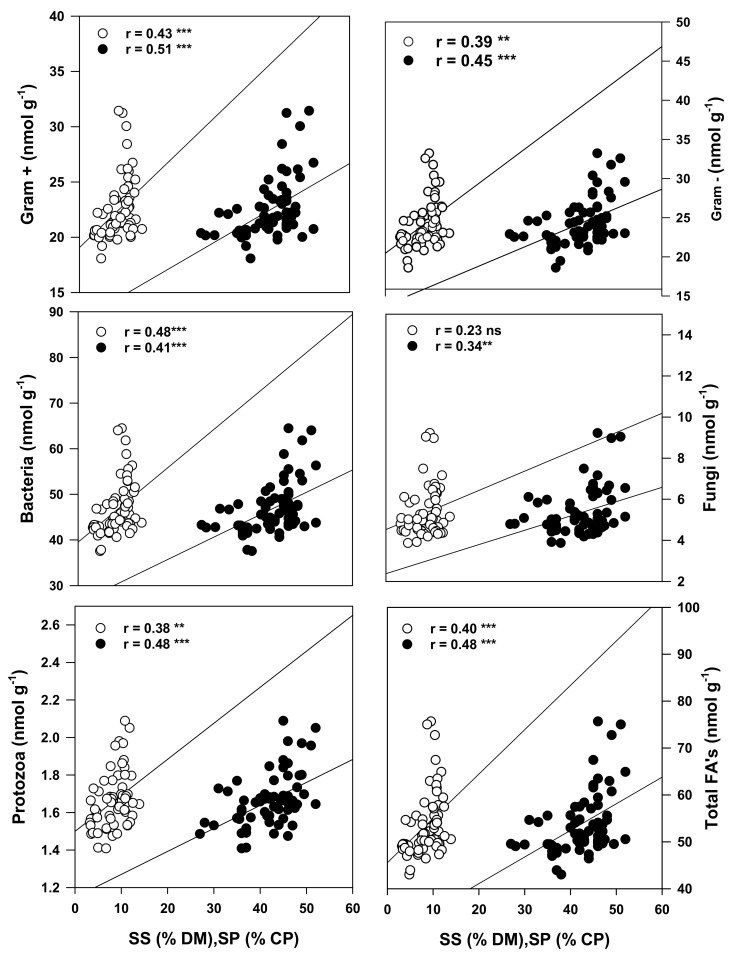
Pearson correlation between SP and SS with microbial PLFAs for different corn–soybean monocropping and intercropping treatments. SP = soluble protein (black circles); SS = soluble sugars (hollow circles); ns = non-significant; * correlation is significant (*p* ≤ 0.05); ** (*p* ≤ 0.01); *** (*p* ≤ 0.001), n = 66 for all parameters.

**Figure 6:**
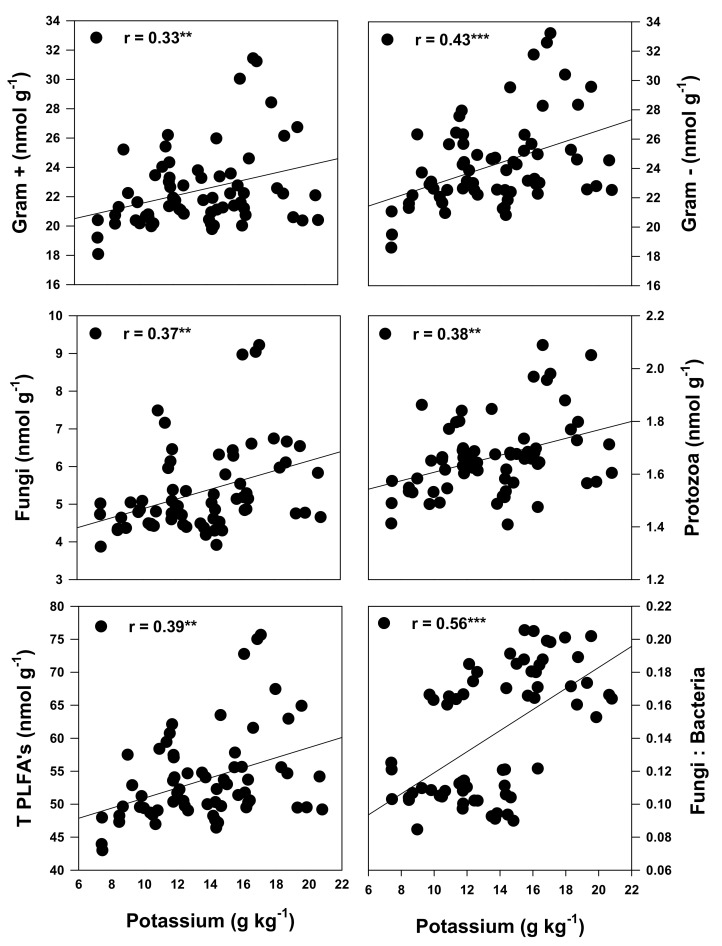
Pearson correlation showing the association between the K content and the active soil microbial community of forage produced from monocropping or intercropping corn and soybeans under cool climatic conditions and the PLFA for different corn–soybean monocropping and intercropping treatments. T PLFA = total phospholipid fatty acids; ns = non-significant; * correlation is significant (*p* ≤ 0.05); ** (*p* ≤ 0.01); *** (*p* ≤ 0.001), n = 66 for all parameters.

The total active microbial population was significantly correlated with SP (r = 0.48) and SS (r = 0.40). Specifically, the SP and SS content were significantly associated with the soil protozoans (r = 0.48 and 0.38), total bacteria (r = 0.41 and 0.48), gram+ (r = 0.51 and 0.43) and gram- (r = 0.45 and 0.39) bacteria populations (Figure 5). The RDA output demonstrated that the mineral nutrients clustered with the monocropped soybeans (Figure 4). The K content of the forage was the only mineral nutrient observed to be significantly correlated with the active soil microbial population. Significant correlations were observed between the fungal (r = 0.37) and protozoan (r = 0.38) populations, the ratio of the fungal: bacteria populations (r = 0.56), the gram+ (r = 0.33) and gram- (r = 0.43) bacteria and the forage K content (Figure 6).

## 4. Discussion

### 4.1. Effect of Intercropping on Forage Quality

Overall, IC significantly increased the CP contents over corn MC in this study. This increase was almost 22% in IC treatments compared with corn MC treatments. The high CP content observed in soybean MC treatments means the increase in CP percentage is due to mixing of soybean with corn as an IC system for producing forage with high nutritional quality when cultivated on podzolic soil under cool climatic conditions in boreal ecosystems. The present results were consistent with other studies done on IC. For example, Baghdadi et al. [14] reported a 30% increase in CP due to IC compared to corn MC. Liu et al. [46] also reported increases in CP ranging from (31 to 59%) due to IC of corn with alfalfa compared to corn MC. This increase in CP content of forage produced following cultivation by IC has also been reported by several other researchers [16,18,47,48,49,50,51].

The higher protein contents obtained from forage produced under IC systems can reduce the requirement for protein supplements in animal feed formulation [49] for livestock production in boreal ecosystems. Other important features of a high quality forage are the concentrations of ADF and NDF [16,47,52,53] present in the forage. During ration formulation, the NDF content is crucial as it is a major determinant in animal forage consumption [16,52]. Forage quality is negatively related to NDF and ADF, and lower values are required for higher quality forage [51,54,55,56]. Our results demonstrate low ADF (−14%) and NDF contents (−6%) in corn–soybean forage produced via IC compared to forage produced in corn monoculture on podzolic soil in boreal climates (Table 4). This is consistent with earlier findings showing that IC corn with soybean decreases the NDF content significantly compared to forage obtained from corn cultivated as a monocrop [23]. Conversely, some studies reported IC had no effect [57] or increased [58] the NDF and ADF levels in the forage produced. The presence of leguminous crops in the IC system reduced the ADF and NDF concentration in the present study (Table 4). Leguminous crops are lower in NDF and ADF compared to grasses [57]. NDF concentration in forage can also be affected by the crop maturity, because maturity changes the levels of hemicellulose, lignin and cellulose, which are essential parts of the plant cell wall [59].

Similar to the NDF values, the WSC level was lower in the forage obtained from the IC treatments compared to corn MC treatments. Legumes, especially soybeans, are known to contain low WSC, but have high protein content [53]. In this study, IC treatments produced WSC lower than corn MC, but higher than soybeans cultivated as MC. These finding are in agreement with the results reported for soybean intercropped with millet [22] and corn intercropped with beans [50,60]. Therefore, increasing the ratio of legumes in cereal–legume IC systems will decrease the WSC [53] to produce high-quality forage as we have observed in our study.

Legumes contain higher ash content than grasses [13]. This was observed in the present study, where both vine and upright soybean cultivated as monocrops had superior ash content compared to corn cultivated as monocrops. Consequently, the ash content was enhanced in the forage mix obtained when silage corn was intercropped with forage soybeans on podzolic soil in boreal climates. The enhanced ash content we observed in the forage obtained from corn intercropped with soybean is in agreement with the reports in the literature for corn–kale, corn–sunflower and corn–runner bean IC systems.

In cool climates, forage energy feeding value is a significant nutritive consideration in beef cattle production, because it is beneficial in improving animal growth and productivity. TDN refers to those nutrients that livestock can utilize and are negatively correlated with the ADF contents in the forage [61]. As the ADF content increases in the forage, TDN decreases, indicating that animals are less able to utilize the nutrients present in the forage. The negative correlation is similar to the present results (Figure 3), where IC treatments generally increased TDN content by 9% compared to corn MC and 13% compared to soybean MC. These finding are consistent with those of Salama and Zeid [62] and Gill and Omokanye [58]. Forage having a range of 55 to 65% TDN is considered a good quality forage. All the IC treatments in the present study were in the range of 55-65% during both growth years. There is a percent rule of thumb for TDN, which states a mature beef cow needs 55%, 60% and 65% energy for mid-pregnancy, late pregnancy and after the calving period, respectively, to maintain her body condition score [63]. The findings from this study suggest the forage produced on podzols in boreal climates from intercropping corn and soybeans appears to meet the rule of thumb TDN requirements for optimized livestock utilization. Furthermore, TDN and CP are critical indices of forage quality and have positive correlation with the forage price [64]. IC also enhanced the NEL (9%), NEM (15%) and NEG (28%) relative to corn MC treatments (Table 5). Lauriault and Kirksey [55] found an increase in NE_L_ of forage produced from pea–wheat or pea–oat IC, while no effect was observed in pea–barley and pea–rye IC systems. Sadeghpour et al. [61,65] also reported increased NE_L_ of forage produced from barley and annual medic IC compared to barley cultivated as a monocrop. CP and sugars are highly digestible nutrients [66], and their contents were significantly improved in the forage produced in the IC treatments when cultivated on podzols in cool climatic conditions. Concomitant with the CP and SS contents, the digestible dry matter increased 7% in the IC treatments evaluated in this study. This indicates that superior DDM content of the forage may be associated with the enhanced level of CP and SS present in the mixed forage obtained from soybean and corn IC [66]. Our findings are consistent with that of Dahmardeh et al. [50], who reported increased DDM in forages obtained from corn intercropped with cowpea compared to forage obtained from corn cultivated in monocultures. Sadeghpour et al. [61,65] reported similar results for DDM when annual medic was intercropped with barley. The higher DDM observed in the intercropped forage mixes may be due to elevated CP and sugar contents [66], because both of these are highly digestible nutrients that are known to contribute to the DDM values in forage based animal feed. Animal productivity is directly related to voluntary intake of forage [67].

Higher voluntary intake means higher DMI, which ultimately results in higher nutrient intake. In this study, we reported the estimated DMI as a measure of potential nutrient intake of the forage produced in cropping systems when cultivated on podzols in boreal climates. The highest DMI was estimated for forages obtained in the soybean MC treatments, and the lowest in corn MC treatments. The IC treatments produced forage with DMI values between soybean and corn cultivated as monocrops in podzolic soil. These results are in agreement with forage obtained from cow pea intercropped with pearl millet and grasses [62]. DMI is higher for leguminous forage than non-leguminous forage [67]. In the present study, there was a negative correlation observed between DMI and NDF, indicating that, as NDF increases, the quality and DMI of forage decreases (Figure 3), consistent with previous findings [68].

RFV is an important index that is used to predict the energy value and forage consumption [16]; it can be calculated from DMI and DDM. Similar to the trends for net energy value, the RFV was also higher (15%) for forages produced in the IC treatments compared to when corn was cultivated as a monocrop. Higher RFVs have been reported for barley intercropped with legumes compared to when barley was cultivated as a monocrop [51,61,62,65]. Forage RFV is known to increase as NDF and ADF values decrease [51]. RFVs for beef cows should be in the range of 90–115, as suggested by Schroeder et al. [69]; this is consistent with the values observed from the forage produced in the IC treatments in our study (Table 5). In the present study, RFV showed significant positive correlations with CP, while significant negative correlations were observed with NDF and ADF; these are consistent with the expected relationships between CP, NDF and ADF, as indicators of superior forage quality [51].

### 4.2. Effect of Intercropping on Forage Mineral Contents

Legumes contain higher total macro and micro nutrients compared to grasses [13]. For instance, Ca is two to three times higher in legumes than all other major forages [13]. Moreover, IC increases forage Ca compared to MC [23]. Higher Ca content has been reported in the literature in IC compared to the corresponding content in MC [58,70]. Conversely, no difference was observed between IC and MC treatments for forage nutrients such as Na, K, P and Mg [23]. The highest Ca contents were recorded in the soybean MC treatments, suggesting that soybean is driving the response. All the IC treatments produced forage that met the required Mg levels for dry gestating (1.2 g/kg) and lactating (2.0 g/kg) beef cows [71]. P and Mg were also in the range required for dry gestating and lactating beef cows [71]. Collectively, these results indicate the IC treatments produced forage with superior mineral content when cultivated on podzols in boreal ecosystems. The content of important micronutrients Zn (30 mg/kg), Fe (50 mg/kg), Cu (10 mg/kg) and Mn (40 mg/kg) observed in the IC treatments in this study is also in the range required for a mature beef cow [71].

### 4.3. Effects of Intercropping on Forage Fatty Acid Composition

In the present study, unsaturated FAs (UFAs), especially PUFAs, were higher in all the treatments compared to saturated FAs (SFAs). This may be a result of the low temperature characteristic of the region during crop cultivation. Plants respond to low growth temperature by increasing UFAs (especially PUFAs) to maintain membrane integrity [72,73,74]. PUFAs play a role in maintaining membrane fluidity in the chloroplast as well as other organelles [75], which is an important plant response to low temperature stress. Forages are often the main source of PUFAs in animal feed, and forages with higher PUFAs can modify the FA profile in meat or dairy products [76,77].

Milk’s FA profile is of prime importance because it is a significant part of the human diet [75] and ultimately depends on the FA profile of the animal’s diet [78]. PUFA contains two major FAs (18:2n-6 and 18:3n-3) in forage. The contribution of 18:2n-6 is almost 91% and 18:3n-3 is 9% in maize silage [79]. Hydrogenation of C18:2n-6 in the rumen of ruminants results into an increase in the concentration of *cis*-9, *trans*-11 C18:2 and *trans*-11 C18:1, which are very valuable for human health [79]. In the present study, an increase (67%) in C18:3n-3 and a decrease (15%) in C18:2n-6 was observed in the forage due to corn–soybean IC compared to corn MC (Table 8). The higher 18:3 and lower 18:2 maybe due to the contribution of the intercropped soybean varieties, because soybeans have higher C18:3n-3 and lower C18:2n-6 FAs compared to corn. Conversely, the corn forage was high in C18:2n-6 but low in C18:3n-3 FAs, which is in agreement with earlier findings [79]. An increase of omega 3 in the intercropped forage may assist in modulating the PUFA profile of the milk produced from animals fed a ration containing this forage in the formulation by increasing the n-3/n-6 and decreasing n-6/n-3 ratios [80].

Dairy cows fed with diets high in 18:3 were observed to produced milk enhanced with PUFAs, especially alpha linoleic acid (ALA) and *c*9, *t*11 conjugated FAs [81]. A high n-6/n-3 ratio can cause coronary heart disease, particularly blood clotting [82]. Furthermore, conjugated linoleic acid (CLA) can be formed from the isomerization of linoleic acid (C18:2n-6) in the rumen, thereby elevating their levels in ruminant food products [83]. In addition, the inclusion of high levels of CLA and other PUFAs in the feed of dairy cows can increase pregnancy rate [84,85]. Milk rich in CLA can be advantageous for human health [86]. Thus, increasing the 18:3 and 18:2 fatty acids in ruminant feed can enhance the availability of CLA in the food products (milk and meat) produced by ruminant animals [87].

Therefore, the production of forage cultivated as intercrops in podzolic soil appears to have enhanced nutritional quality and could be of relevance in improving overall animal production and quality of the animal products obtained, particularly in animals reared in northern climates or cool climatic regions around the world.

### 4.4. Relationship between Soil Health Status and Forage Nutritional Quality

All the forage quality parameters showed a significant (*p* ≤ 0.05) positive correlation with the major forage FA contents, while a negative correlation was observed between NDF, ADF, WSC, SS, SP and total and major FA contents (Table 9, Figure 2). This showed that FA contents are negatively correlated with plant maturity [61,76,88]. As the plant matures, the ADF and NDF content increased, while the FA level decreased in forage produced in podzolic soil under cool climatic conditions. The FA content decreases with plant maturity, due to low leaf/stem ratio, flower initiation and leaf senescence [76,88]. ADF and NDF are significantly (*p* ≤ 0.001) but negatively correlated with RFV. However, the significant positive correlation between RFV and CP showed that the forage contains higher CP contents and is of higher quality than a forage with high NDF and ADF content. Fiber content (ADF and NDF) can decrease animal feed intake and TDN in the forage, because DMI is negatively correlated with NDF [68], while the TDN is negatively correlated with ADF [61] this is consistent with the observations in our study (Figure 3). The soil microbial community composition showed a significant positive correlation with the K contents of the forage (Figure 6). These findings indicate that the increased bioaccumulations of K in forage crops cultivated on podzolic soil in boreal climates may be due to higher mineralization of nutrients in the soil by the active soil microbial community (bacteria, protozoans and fungi).

The microbial population was also observed to be significantly associated with the SP and SS contents in the forage (soybean and corn) produced as intercrops or monocultures (Figure 5). These significant relationships suggest for the first time that the active microbial population, particularly the protozoans and gram+ and gram− bacteria, appears to enhance the nutritive value of corn and soybean forage produced in podzols as either monocrops or intercrops in northern or cool climates.

## 5. Conclusions

Overall, the current study findings demonstrate that IC was superior to corn MC treatments in terms of enhancing the overall forage quality and mineral and FA composition. Specifically, the CP, DMI, DDM, TDN, NEL, NEM and NEG values were higher in IC compared to corn MC, while ADF and NDF values were reduced when silage corn was intercropped with forage soybeans. The observed trends indicate that soybean IC with C2 performed better compared to intercropping with C1 in terms of forage quality. When comparing soybean varieties, the upright soybeans generally performed better than vine soybeans. IC resulted in higher omega 3/omega 6 ratios and forage minerals. The active microbial community structure is significantly associated with the K, SP and SS content in forage. The results of this study suggest that forage soybean inclusion in exiting silage corn cropping systems would sustainably produce forage with improved nutritional composition. This work will be of value to animal and agriculture production in northern or boreal climates, particularly in the context of improving food security to meet the anticipated future growth in global populations, including those in northern climates or boreal regions of the world.

## Figures and Tables

**Table 1:** Soil chemical properties before the experiment for 2016 and 2017 growing seasons.

pH	Organic Matter (%)	N (%)	P ppm	K ppm	Na ppm	Zn ppm	Mn ppm	S ppm	Fe ppm	Ca ppm	Mg ppm
**2016**											
6.4	2.98	0.2	81	38	7	0.6	18	14	150	1256	265
**2017**											
6.8	3.38	0.2	68	35	5	1.0	10	17	233	1426	322

**Table 2:** The average maximum and minimum temperature and total rainfall during 2016 and 2017 growing seasons.

	Growing Season 2016			Growing Season 2017		
Month	Rainfall (mm)	Tmax (°C)	Tmin (°C)	Rainfall (mm)	Tmax (°C)	Tmin (°C)
May (15–30)	47	16.3	2.8	50	13.5	−0.1
June	148	19.1	6.1	80	18.4	6.0
July	82	23.0	9.5	41	23.8	8.5
August	139	22.4	10	85	22.9	8.1
September	133	16.3	5.4	156	17.2	5.2
October (1–25)	182	13.0	0.8	78	12.6	1.6
	731	18.3	5.7	490	18.1	4.9

**Table 3:** Microwave digestion program used for plant tissue digestion prior to mineral analysis.

Steps	Ramp (mm:ss)	Temperature (°C)	Hold (mm:ss)
1	10:00	140	5:00
2	1:00	160	15:00

**Table 4:** The effects of corn and soybean intercropping or monocropping treatments on forage protein, fiber, sugar and ash content during the 2016 and 2017 growing seasons.

Growing Seasons (2016–17)								
Treatments	CP (%DM)	AP (%DM)	SP (%CP)	ADF (%DM)	NDF (%DM)	WSC (%DM)	SS (%DM)	Ash (%DM)
C1	11.1 ± 0.30 ^de^	10.3 ± 0.29 ^ef^	39.8 ± 1.48 ^cdef^	37.7 ± 1.83 ^a^	57.7 ± 0.61 ^a^	13.9 ± 0.66 ^b^	6.1 ± 1.04 ^d^	6.8 ± 1.41 ^de^
C2	10.3 ± 0.21 ^e^	9.7 ± 0.17 ^f^	40.8 ± 1.74 ^bcde^	36.8 ± 1.18 ^ab^	57.7 ± 0.16 ^a^	17.3 ± 1.49 ^a^	7.5 ± 1.29 ^cd^	6.0 ± 0.49 ^e^
S1	19.4 ± 1.16 ^a^	17.3 ± 0.92 ^a^	35.7 ± 1.54 ^f^	34.7 ± 1.36 ^abcde^	41.4 ± 2.64 ^f^	7.0 ± 0.53 ^d^	6.0 ± 0.47 ^d^	14.0 ± 1.48 ^ab^
S2	18.1 ± 0.74 ^ab^	15.9 ± 0.42 ^b^	38.5 ± 1.78 ^def^	35.1 ± 0.98 ^abcd^	43.1 ± 2.07 ^ef^	7.7 ± 0.33 ^d^	5.7 ± 0.80 ^d^	12.9 ± 1.14 ^bc^
S3	17.6 ± 0.51 ^b^	14.9 ± 0.50 ^b^	36.2 ± 3.53 ^ef^	35.6 ± 1.01 ^abc^	47.3 ± 1.39 ^de^	5.4 ± 0.86 ^d^	5.9 ± 1.14 ^d^	17.9 ± 2.60 ^a^
S1C1	12.5 ± 0.21 ^cd^	11.3 ± 0.21 ^de^	46.8 ± 1.47 ^a^	33.6 ± 1.11 ^bcde^	55.4 ± 0.97 ^ab^	11.3 ± 0.76 ^c^	10.2 ± 0.36 ^ab^	10.1 ± 1.44 ^bcd^
S2C1	13.1 ± 0.24 ^cd^	11.9 ± 0.19 ^cd^	44.3 ± 0.80 ^abc^	33.1 ± 0.94 ^cdef^	55.4 ± 1.01 ^ab^	11.6 ± 1.05 ^c^	10.5 ± 0.24 ^ab^	8.8 ± 0.91 ^cde^
S3C1	12.4 ± 0.30 ^cd^	11.7 ± 0.21 ^cd^	47.8 ± 1.01 ^a^	32.5 ± 0.79 ^cdef^	56.3 ± 1.02 ^ab^	12.3 ± 0.99 ^bc^	11.4 ± 0.53 ^a^	8.9 ± 1.16 ^cde^
S1C2	13.8 ± 0.38 ^c^	12.7 ± 0.38 ^c^	47.4 ± 1.04 ^a^	31.2 ± 1.32 ^ef^	52.6 ± 1.00 ^bc^	12.0 ± 0.56 ^bc^	11.0 ± 0.47 ^ab^	8.7 ± 1.30 ^de^
S2C2	13.3 ± 0.48 ^c^	12.5 ± 0.56 ^cd^	41.7 ± 1.33 ^bcd^	29.9 ± 1.82 ^f^	50.9 ± 2.28 ^cd^	12.0 ± 0.36 ^bc^	9.0 ± 0.59 ^bc^	10.4 ± 1.73 ^bcd^
S3C2	13.3 ± 0.28 ^c^	11.9 ± 0.31 ^cd^	45.1 ± 1.13 ^ab^	31.7 ± 0.98 ^def^	54.2 ± 1.01 ^abc^	12.4 ± 0.38 ^bc^	11.3 ± 0.30 ^a^	9.4 ± 1.24 ^cde^
Average	14.1 ± 0.38	12.7 ± 0.30	42.2 ± 1.70	33.8 ± 0.45	52.0 ± 0.79	11.2 ± 0.46	8.6 ± 0.35	10.4 ± 0.57
Mono-(US)	18.8 ± 0.68	16.6 ± 0.53	37.1 ± 1.20	34.9 ± 0.80	42.3 ± 1.62	7.3 ± 0.32 ^A^	5.9 ± 0.45	13.4 ± 0.91 ^B^
Mono-(VS)	17.6 ± 0.51	14.9 ± 0.50	36.2 ± 3.53	35.6 ± 1.01	47.3 ± 1.39	5.4 ± 0.86 ^B^	5.9 ± 1.14	17.9 ± 2.60 ^A^
Inter-(US)	13.1 ± 0.19	12.1 ± 0.20	45.1 ± 0.73	31.9 ± 0.69	53.6 ± 0.78	11.7 ± 0.34	10.2 ± 0.25 ^B^	9.5 ± 0.66
Inter-(VS)	12.8 ± 0.24	11.8 ± 0.18	46.4 ± 0.83	32.1 ± 0.61	55.2 ± 0.75	12.4 ± 0.51	11.4 ± 0.29 ^A^	9.1 ± 0.81
Mono-S	18.4 ± 0.49 ^A^	15.3 ± 0.40 ^A^	36.8 ± 1.36 ^C^	35.1 ± 0.62 ^A^	43.9 ± 1.28 ^C^	6.7 ± 0.41 ^C^	5.9 ± 0.46 ^B^	14.9 ± 1.13 ^A^
Mono-C	10.7 ± 0.20 ^C^	10.2 ± 0.33 ^C^	40.3 ± 1.10 ^B^	37.3 ± 1.02 ^A^	57.7 ± 0.30 ^B^	15.6 ± 0.93 ^A^	6.8 ± 0.82 ^B^	6.4 ± 0.72 ^C^
Inter-(C + S)	13.0 ± 0.15 ^B^	12.1 ± 0.28 ^B^	45.5 ± 0.56 ^A^	32.0 ± 0.50 ^B^	54.1 ± 0.58 ^A^	11.9 ± 0.29 ^B^	10.6 ± 0.21 ^A^	9.4 ± 0.51 ^B^

Crude protein = CP; Available Protein = AP; Soluble Protein = SP; Acid Detergent Fiber = ADF; Neutral Detergent Fiber = NDF; Water Soluble Carbohydrates = WSC; Simple Sugars = SS; Values are means ± standard errors. Mean values in each column followed by the same letter are not significantly different at (LSD, 0.05). C1 = Yukon-R; C2 = DKC-2628; S1 = Big Fellow RR; S2 = Game Keeper RR; S3 = Kester’s Bob White Trailing Soybean; S1C1 = Big Fellow RR + Yukon-R; S2C1 = Game Keeper RR + Yukon-R; S3C1 = Kester’s Bob White Trailing Soybean + Yukon-R; S1C2 = Big Fellow RR + DKC-2628; S2C2 = Game Keeper RR + DKC-2628; S3C2 = Kester’s Bob White Trailing Soybean + DKC-2628. Mono C = Monocropping Corn; Mono S = Monocropping Soybean; Mono-(US) = Monocropping Upright Soybean; Mono-(VS) = Monocropping Vine Soybean; Inter-(US) = Upright Soybean Intercropped with Corn; Inter-(VS) = Vine Soybean Intercropped with Corn.

**Table 5:** The effects of intercropping or monocropping treatments on the total digestible nutrients (TDN), net energy for lactation (NEL), net energy for maintenance (NEM), net energy of gain (NEG), digestible dry matter (DDM), dry matter intake (DMI) and relative feed value (RFV) of forage cultivated under cool climatic conditions during the 2016 and 2017 growing seasons.

	Growing Seasons (2016–17)						
Treatments	TDN %	NEL (Mcal/kg)	NEM (Mcal/kg)	NEG (Mcal/kg)	DDM %	DMI ^3^	RFV %
C1	55.6 ± 1.9 ^ab^	1.17 ± 0.04 ^bcd^	1.08 ± 0.05 ^ab^	0.53 ± 0.05 ^ab^	59.5 ± 1.37 ^f^	2.06 ± 0.02 ^d^	95 ± 2.8 ^f^
C2	54.2 ± 1.5 ^b^	1.13 ± 0.04 ^cd^	1.14 ± 0.05 ^b^	0.48 ± 0.05 ^b^	60.2 ± 0.92 ^ef^	2.08 ± 0.00 ^d^	97 ± 1.5 ^f^
S1	56.7 ± 1.9 ^ab^	1.30 ± 0.03 ^a^	1.17 ± 0.04 ^ab^	0.60 ± 0.03 ^ab^	61.9 ± 1.06 ^bcdef^	2.99 ± 0.17 ^a^	144 ± 10.1 ^a^
S2	54.7 ± 1.4 ^ab^	1.30 ± 0.03 ^a^	1.14 ± 0.04 ^ab^	0.59 ± 0.03 ^ab^	61.6 ± 0.77 ^cdef^	2.80 ± 0.08 ^ab^	134 ± 5.6 ^ab^
S3	47.8 ± 2.0 ^c^	1.08 ± 0.05 ^d^	0.86 ± 0.07 ^c^	0.32 ± 0.07 ^c^	61.2 ± 0.78 ^def^	2.64 ± 0.04 ^b^	125 ± 3.4 ^bc^
S1C1	59.8 ± 1.7 ^a^	1.27 ± 0.04 ^ab^	1.22 ± 0.07 ^a^	0.64 ± 0.06 ^ab^	62.8 ± 0.86 ^bcde^	2.18 ± 0.03 ^d^	106 ± 3.0 ^ef^
S2C1	59.2 ± 2.0 ^ab^	1.26 ± 0.04 ^ab^	1.22 ± 0.06 ^a^	0.65 ± 0.05 ^a^	63.1 ± 0.73 ^abcd^	2.20 ± 0.03 ^d^	108 ± 2.6 ^ef^
S3C1	59.7 ± 1.3 ^ab^	1.22 ± 0.03 ^abc^	1.21 ± 0.05 ^ab^	0.64 ± 0.04 ^ab^	63.6 ± 0.61 ^abcd^	2.10 ± 0.04 ^d^	104 ± 2.8 ^ef^
S1C2	60.2 ± 2.4 ^a^	1.26 ± 0.05 ^ab^	1.22 ± 0.08 ^a^	0.66 ± 0.08 ^a^	64.6 ± 1.03 ^ab^	2.25 ± 0.02 ^cd^	113 ± 2.5 ^cde^
S2C2	60.2 ± 2.9 ^a^	1.27 ± 0.05 ^ab^	1.23 ± 0.07 ^a^	0.66 ± 0.07 ^a^	65.6 ± 1.42 ^a^	2.41 ± 0.10 ^c^	123 ± 7.7 ^bcd^
S3C2	59.2 ± 2.1 ^ab^	1.24 ± 0.05 ^abc^	1.19 ± 0.07 ^ab^	0.64 ± 0.07 ^ab^	64.2 ± 0.76 ^abc^	2.23 ± 0.05 ^cd^	111 ± 3.5 ^de^
Average	57.0 ± 0.7	1.22 ± 0.01	1.14 ± 0.02	0.58 ± 0.02	62.6 ± 0.35	2.36 ± 0.04	115 ± 2.3
Mono-(US)	55.7 ± 1.2 ^A^	1.30 ± 0.02 ^A^	1.15 ± 0.03 ^A^	0.59 ± 0.02 ^A^	61.7 ± 0.62	2.90 ± 0.09	139 ± 5.7
Mono-(VS)	47.8 ± 2.0 ^B^	1.08 ± 0.05 ^B^	0.86 ± 0.07 ^B^	0.32 ± 0.07 ^B^	61.2 ± 0.78	2.64 ± 0.04	125 ± 4.7
Inter-(US)	59.8 ± 1.1	1.26 ± 0.02	1.22 ± 0.03	0.65 ± 0.03	64.0 ± 0.54	2.26 ± 0.03	112 ± 2.5
Inter-(VS)	59.4 ± 1.2	1.23 ± 0.03	1.20 ± 0.04	0.64 ± 0.04	63.9 ± 0.48	2.17 ± 0.03	107 ± 2.4
Mono-S	53.1 ± 1.3 ^B^	1.22 ± 0.03 ^AB^	1.05 ± 0.04 ^B^	0.50 ± 0.04 ^B^	61.5 ± 0.48 ^B^	2.81 ± 0.07 ^A^	135 ± 4.5 ^A^
Mono-C	54.9 ± 1.2 ^B^	1.15 ± 0.03 ^B^	1.06 ± 0.04 ^B^	0.51 ± 0.03 ^B^	59.9 ± 0.79 ^B^	2.07 ± 0.01 ^C^	96 ± 1.2 ^C^
Inter-(C + S)	59.7 ± 0.8 ^A^	1.25 ± 0.02 ^A^	1.21 ± 0.02 ^A^	0.65 ± 0.02 ^A^	64.0 ± 0.39 ^A^	2.23 ± 0.03 ^B^	111 ± 1.9 ^B^

^3^ = %BW. Values are means ± standard errors. Mean values in each column followed by the same letter are not significantly different at (LSD, 0.05). C1 = Yukon-R; C2 = DKC-2628; S1 = Big Fellow RR; S2 = Game Keeper RR; S3 = Kester’s Bob White Trailing Soybean; S1C1 = Big Fellow RR + Yukon-R; S2C1 = Game Keeper RR + Yukon-R; S3C1 = Kester’s Bob White Trailing Soybean + Yukon-R; S1C2 = Big Fellow RR + DKC26-28; S2C2 = Game Keeper RR + DKC26-28; S3C2 = Kester’s Bob White Trailing Soybean + DKC26-28. Mono C = Monocropping Corn; Mono S = Monocropping Soybean; Mono-(US) = Monocropping Upright Soybean; Mono-(VS) = Monocropping Vine Soybean; Inter-(US) = Upright Soybean Intercropped with Corn; Inter-(VS) = Vine Soybean Intercropped with Corn.

**Table 6:** Macro-nutrient content of forage obtained from corn and soybean cultivated as monocrops and intercrops during the 2016 and 2017 growing seasons.

	Growing Seasons (2016–17)				
Treatments	Ca (g/kg)	P (g/kg)	Mg (g/kg)	K (g/kg)	Na (mg/kg)
C1	0.14 ± 0.03 ^e^	2.10 ± 0.16 ^e^	2.93 ± 0.19 ^ef^	13.0 ± 1.12 ^cd^	26.5 ± 8.69 ^de^
C2	0.16 ± 0.03 ^e^	2.14 ± 0.15 ^e^	2.64 ± 0.15 ^f^	9.4 ± 0.88 ^e^	13.4 ± 3.12 ^e^
S1	1.42 ± 0.05 ^b^	3.61 ± 0.11 ^a^	7.07 ± 0.11 ^a^	16.8 ± 1.11 ^ab^	51.7 ± 8.50 ^ab^
S2	1.60 ± 0.08 ^a^	3.55 ± 0.07 ^ab^	7.02 ± 0.28 ^a^	17.5 ± 1.19 ^a^	59.2 ± 4.19 ^a^
S3	1.01 ± 0.04 ^c^	2.85 ± 0.39 ^cd^	6.27 ± 0.14 ^b^	12.3 ± 0.95 ^cde^	54.3 ± 6.36 ^ab^
S1C1	0.37 ± 0.06 ^d^	2.38 ± 0.16 ^de^	3.29 ± 0.31 ^def^	14.2 ± 1.12 ^bc^	41.8 ± 7.02 ^bcd^
S2C1	0.43 ± 0.04 ^d^	2.76 ± 0.25 ^cd^	3.55 ± 0.13 ^de^	13.7 ± 0.89 ^bcd^	51.4 ± 7.00 ^ab^
S3C1	0.45 ± 0.03 ^d^	2.62 ± 0.13 ^cde^	4.28 ± 0.16 ^c^	14.6 ± 0.77 ^abc^	41.2 ± 4.22 ^bcd^
S1C2	0.36 ± 0.06 ^d^	2.71 ± 0.17 ^cd^	3.66 ± 0.43 ^cd^	14.0 ± 2.14 ^bc^	31.1 ± 3.44 ^cd^
S2C2	0.45 ± 0.03 ^d^	3.04 ± 0.08 ^bc^	3.72 ± 0.31 ^cd^	13.7 ± 0.88 ^bcd^	46.8 ± 2.88 ^abc^
S3C2	0.40 ± 0.03 ^d^	2.82 ± 0.13 ^cd^	3.51 ± 0.19 ^de^	10.5 ± 0.84 ^de^	40.7 ± 4.25 ^bcd^
Average	0.62 ± 0.06	2.78 ± 0.08	4.36 ± 0.20	13.6 ± 0.42	41.7 ± 2.27
Mono-(US)	1.51 ± 0.05 ^B^	3.58 ± 0.06 ^A^	7.05 ± 0.14 ^A^	17.2 ± 0.78 ^A^	55.5 ± 4.66
Mono-(VS)	1.01 ± 0.04 ^A^	2.85 ± 0.39 ^B^	6.27 ± 0.14 ^B^	12.3 ± 0.95 ^B^	54.3 ± 6.36
Inter-(US)	0.40 ± 0.02	2.72 ± 0.10	3.55 ± 0.15	13.9 ± 0.64	42.8 ± 2.98
Inter-(VS)	0.43 ± 0.02	2.72 ± 0.09	3.89 ± 0.16	12.6 ± 0.82	41.0 ± 2.86
Mono-S	1.34 ± 0.07 ^A^	3.33 ± 0.15 ^A^	6.79 ± 0.14 ^A^	15.5 ± 0.82 ^A^	55.1 ± 3.65 ^A^
Mono-C	0.15 ± 0.02 ^C^	2.12 ± 0.11 ^B^	2.79 ± 0.12 ^C^	11.2 ± 0.87 ^C^	20.0 ± 4.82 ^C^
Inter-(C + S)	0.41 ± 0.02 ^B^	2.72 ± 0.07 ^A^	3.67 ± 0.12 ^B^	13.5 ± 0.51 ^B^	42.2 ± 2.19 ^B^

Values are means ± standard errors. Mean values in each column followed by the same letter are not significantly different at (LSD, 0.05) C1 = Yukon-R; C2 = DKC26-28; S1 = Big Fellow RR; S2 = Game Keeper RR; S3 = Kester’s Bob White Trailing Soybean; S1C1 = Big Fellow RR + Yukon-R; S2C1 = Game Keeper RR + Yukon-R; S3C1 = Kester’s Bob White Trailing Soybean + Yukon-R; S1C2 = Big Fellow RR + DKC26-28; S2C2 = Game Keeper RR + DKC26-28; S3C2 = Kester’s Bob White Trailing Soybean + DKC26-28. Mono C = Monocropping Corn; Mono S = Monocropping Soybean; Mono-(US) = Monocropping Upright Soybean; Mono-(VS) = Monocropping Vine Soybean; Inter-(US) = Upright Soybean Intercropped with Corn; Inter-(VS) = Vine Soybean Intercropped with Corn.

**Table 7:** Micronutrient content of forage obtained from corn and soybean cultivated as monocrops and intercrops during the 2016 and 2017 growing seasons.

Growing Seasons (2016–17)						
Treatments	Zn (mg/kg)	Fe (mg/kg)	B (mg/kg)	Mn (mg/kg)	Cu (mg/kg)	Co (mg/kg)
C1	17.5 ± 0.57 ^cde^	307 ± 51.3 ^b^	5.2 ± 0.56 ^e^	74.6 ± 20.3 ^cde^	10.1 ± 1.86 ^cde^	0.92 ± 0.37 ^ab^
C2	10.8 ± 1.23 ^e^	247 ± 54.2 ^b^	5.5 ± 0.70 ^e^	56.9 ± 13.5 ^e^	7.3 ± 1.49 ^e^	0.36 ± 0.14 ^b^
S1	35.4 ± 1.71 ^a^	1092 ± 213.6 ^a^	29.5 ± 1.85 ^a^	206.0 ± 43.7 ^a^	14.7 ± 1.58 ^abc^	1.85 ± 0.54 ^a^
S2	17.1 ± 1.97 ^de^	1244 ± 318.3 ^a^	32.1 ± 1.13 ^a^	142.4 ± 8.9 ^abc^	16.8 ± 1.14 ^a^	1.07 ± 0.16 ^ab^
S3	31.4 ± 1.90 ^ab^	963 ± 92.5 ^a^	22.8 ± 1.56 ^b^	164.9 ± 2.6 ^ab^	16.4 ± 2.58 ^ab^	1.93 ± 0.75 ^a^
S1C1	26.5 ± 6.86 ^abcd^	474 ± 74.3 ^b^	10.2 ± 1.69 ^cd^	83.3 ± 18.8 ^cde^	14.1 ± 3.38 ^abcd^	0.97 ± 0.39 ^ab^
S2C1	26.6 ± 2.96 ^abc^	386 ± 39.2 ^b^	9.0 ± 0.56 ^cd^	85.6 ± 14.0 ^cde^	12.0 ± 1.25 ^abcde^	0.78 ± 0.24 ^ab^
S3C1	19.6 ± 1.07 ^cde^	377 ± 36.6 ^b^	7.2 ± 0.74 ^de^	128.8 ± 35.4 ^bcd^	11.5 ± 1.77 ^bcde^	1.25 ± 0.49 ^ab^
S1C2	18.7 ± 0.55 ^cde^	372 ± 42.3 ^b^	7.6 ± 0.38 ^de^	58.4 ± 8.3 ^de^	9.0 ± 1.50 ^de^	0.50 ± 0.18 ^b^
S2C2	25.4 ± 2.68 ^bcd^	524 ± 60.3 ^b^	11.0 ± 0.50 ^c^	119.8 ± 25.0 ^bcde^	9.4 ± 1.10 ^de^	1.47 ± 0.53 ^ab^
S3C2	33.6 ± 6.59 ^ab^	495 ± 26.8 ^b^	9.2 ± 0.69 ^cd^	93.8 ± 11.1 ^bcde^	10.9 ± 0.83 ^cde^	0.77 ± 0.24 ^ab^
Average	23.9 ± 1.30	589 ± 53.6	13.6 ± 1.19	110.4 ± 8.8	12.0 ± 0.62	1.08 ± 0.13
Mono-(US)	26.2 ± 3.03	1168 ± 184.2	30.8 ± 1.11 ^A^	174.2 ± 23.4	15.8 ± 0.98	1.46 ± 0.29
Mono-(VS)	31.4 ± 1.90	963 ± 92.5	22.8 ± 1.56 ^B^	164.9 ± 39.3	16.4 ± 2.58	1.93 ± 0.75
Inter-(US)	24.3 ± 1.98	439 ± 29.1	9.5 ± 0.51	86.8 ± 9.4	11.1 ± 1.04	0.93 ± 0.18
Inter-(VS)	26.6 ± 3.82	436 ± 28.0	8.2 ± 0.57	111.3 ± 18.5	11.2 ± 0.94	1.01 ± 0.27
Mono-S	27.9 ± 2.16 ^A^	1099 ± 126.6 ^A^	28.2 ± 1.26 ^A^	171.1 ± 19.7 ^A^	16.0 ± 1.03 ^A^	1.62 ± 0.31 ^A^
Mono-C	14.1 ± 1.21 ^B^	277 ± 36.7 ^B^	5.4 ± 0.43 ^C^	65.7 ± 11.9 ^B^	8.7 ± 1.21 ^B^	0.64 ± 0.21 ^B^
Inter-(C + S)	25.1 ± 1.81 ^A^	438 ± 21.3 ^B^	9.0 ± 0.40 ^B^	95.0 ± 8.8 ^B^	11.1 ± 0.75 ^B^	0.96 ± 0.15 ^B^

Values are means ± standard errors. Mean values in each column followed by the same letter are not significantly different at (LSD, 0.05). C1 = Yukon-R; C2 = DKC26-28; S1 = Big Fellow RR; S2 = Game Keeper RR; S3 = Kester’s Bob White Trailing Soybean; S1C1 = Big Fellow RR + Yukon-R; S2C1 = Game Keeper RR + Yukon-R; S3C1 = Kester’s Bob White Trailing Soybean + Yukon-R; S1C2 = Big Fellow RR + DKC26-28; S2C2 = Game Keeper RR + DKC26-28; S3C2 = Kester’s Bob White Trailing Soybean + DKC26-28. Mono C = Monocropping Corn; Mono S = Monocropping Soybean; Mono-(US) = Monocropping Upright Soybean; Mono-(VS) = Monocropping Vine Soybean; Inter-(US) = Upright Soybean Intercropped with Corn; Inter-(VS) = Vine Soybean Intercropped with Corn.

**Table 8:** Plant fatty acids profile (g/kg DM) of corn and soybean cultivated as monocrops and as intercrops during the 2016 and 2017 growing seasons.

Growing Seasons (2016–17)										
Treatments	C16-0	C16-1n-7	C18-0	C18-1n-9cis	C18-2n-6	C18:3n-3	Total FA	SFA %	MUFA %	PUFA %
C1	1.73 ± 0.03 ^f^	0.12 ± 0.03 ^b^	0.15 ± 0.02 ^c^	0.98 ± 0.11 ^cd^	2.54 ± 0.48 ^ab^	0.46 ± 0.03 ^d^	5.98 ± 0.33 ^d^	31.8 ± 1.58 ^cd^	19.2 ± 3.38 ^ab^	49.0 ± 4.93
C2	1.75 ± 0.08 ^ef^	0.12 ± 0.03 ^b^	0.17 ± 0.03 ^c^	0.93 ± 0.09 ^cd^	2.90 ± 0.47 ^a^	0.50 ± 0.01 ^cd^	6.37 ± 0.25 ^cd^	30.3 ± 1.34 ^d^	16.9 ± 2.52 ^ab^	52.9 ± 3.63
S1	2.30 ± 0.10 ^a^	0.42 ± 0.04 ^a^	0.47 ± 0.04 ^a^	0.66 ± 0.12 ^d^	1.84 ± 0.19 ^b^	1.84 ± 0.09 ^a^	7.53 ± 0.37 ^ab^	36.8 ± 0.69 ^a^	15.0 ± 2.73 ^b^	48.2 ± 3.04
S2	1.98 ± 0.06 ^bc^	0.38 ± 0.04 ^a^	0.40 ± 0.05 ^ab^	0.50 ± 0.07 ^d^	1.59 ± 0.11 ^b^	1.58 ± 0.04 ^a^	6.43 ± 0.30 ^cd^	37.0 ± 0.21 ^a^	14.2 ± 2.31 ^b^	48.8 ± 2.29
S3	1.81 ± 0.04 ^def^	0.38 ± 0.09 ^a^	0.35 ± 0.02 ^b^	0.55 ± 0.05 ^d^	1.63 ± 0.32 ^b^	1.55 ± 0.05 ^a^	6.27 ± 0.43 ^cd^	35.4 ± 2.64 ^ab^	15.9 ± 3.31 ^ab^	48.7 ± 5.92
S1C1	1.78 ± 0.05 ^def^	0.17 ± 0.04 ^b^	0.19 ± 0.02 ^c^	1.26 ± 0.11 ^bc^	2.36 ± 0.43 ^ab^	0.64 ± 0.01 ^bcd^	6.39 ± 0.30 ^cd^	31.1 ± 1.12 ^cd^	23.1 ± 3.31 ^ab^	45.8 ± 4.40
S2C1	1.87 ± 0.04 ^cdef^	0.17 ± 0.04 ^b^	0.22 ± 0.03 ^c^	1.36 ± 0.23 ^abc^	2.47 ± 0.38 ^ab^	0.75 ± 0.03 ^bcd^	6.84 ± 0.09 ^bc^	30.6 ± 0.68 ^cd^	22.6 ± 4.12 ^ab^	46.8 ± 4.72
S3C1	1.82 ± 0.05 ^cdef^	0.15 ± 0.03 ^b^	0.23 ± 0.01 ^c^	0.96 ± 0.15 ^cd^	2.03 ± 0.29 ^ab^	0.91 ± 0.01 ^b^	6.10 ± 0.25 ^cd^	33.9 ± 0.79 ^abc^	18.9 ± 3.59 ^ab^	47.3 ± 4.29
S1C2	2.05 ± 0.04 ^b^	0.20 ± 0.05 ^b^	0.23 ± 0.03 ^c^	1.85 ± 0.34 ^a^	2.56 ± 0.30 ^ab^	0.78 ± 0.03 ^bcd^	7.67 ± 0.18 ^a^	29.7 ± 0.27 ^d^	26.2 ± 4.45 ^a^	44.1 ± 4.68
S2C2	1.89 ± 0.03 ^bcde^	0.14 ± 0.03 ^b^	0.20 ± 0.02 ^c^	1.38 ± 0.18 ^abc^	1.96 ± 0.30 ^ab^	0.84 ± 0.01 ^bc^	6.41 ± 0.17 ^cd^	32.7 ± 0.92 ^bcd^	24.1 ± 3.71 ^ab^	43.2 ± 4.54
S3C2	1.94 ± 0.05 ^bcd^	0.17 ± 0.04 ^b^	0.21 ± 0.03 ^c^	1.57 ± 0.31 ^ab^	2.51 ± 0.44 ^ab^	0.92 ± 0.01 ^b^	7.32 ± 0.12 ^ab^	29.5 ± 1.31 ^d^	24.1 ± 5.01 ^ab^	46.4 ± 6.29
Average	1.90 ± 0.03	0.22 ± 0.03	0.26 ± 0.01	1.09 ± 0.07	2.22 ± 0.11	0.98 ± 0.09	6.67 ± 0.10	32.6 ± 0.47	20.0 ± 1.11	47.4 ± 1.30
Mono-(US)	2.14 ± 0.07 ^A^	0.40 ± 0.09	0.44 ± 0.03	0.58 ± 0.07	1.72 ± 0.11	1.71 ± 0.14	6.98 ± 0.28	36.9 ± 0.35	14.6 ± 1.71	48.5 ± 1.82
Mono-(VS)	1.81 ± 0.04 ^B^	0.38 ± 0.01	0.35 ± 0.02	0.55 ± 0.05	1.63 ± 0.32	1.55 ± 0.26	6.27 ± 0.43	35.4 ± 2.64	15.9 ± 3.31	48.7 ± 5.92
Inter-(US)	1.90 ± 0.03	0.17 ± 0.02	0.21 ± 0.01	1.46 ± 0.12	2.34 ± 0.17	0.75 ± 0.02 ^B^	6.83 ± 0.14	31.0 ± 0.44	24.0 ± 1.85	45.0 ± 2.16
Inter-(VS)	1.88 ± 0.04	0.16 ± 0.02	0.22 ± 0.02	1.27 ± 0.19	2.27 ± 0.26	0.92 ± 0.06 ^A^	6.71 ± 0.23	31.7 ± 0.98	21.5 ± 3.04	46. ± 3.63
Mono-S	2.03 ± 0.06 ^A^	0.39 ± 0.03 ^A^	0.41 ± 0.02 ^A^	0.57 ± 0.05 ^C^	1.69 ± 0.12 ^B^	1.66 ± 0.13 ^A^	6.74 ± 0.24 ^AB^	36.4 ± 0.88 ^A^	15.0 ± 1.53 ^B^	48.6 ± 2.20
Mono-C	1.74 ± 0.04 ^C^	0.12 ± 0.02 ^B^	0.16 ± 0.02 ^C^	0.95 ± 0.13 ^B^	2.72 ± 0.30 ^A^	0.48 ± 0.02 ^C^	6.17 ± 0.20 ^B^	31.0 ± 1.02 ^B^	18.1 ± 2.04 ^AB^	50.9 ± 2.98
Inter-(C + S)	1.89 ± 0.02 ^B^	0.17 ± 0.01 ^B^	0.21 ± 0.01 ^B^	1.40 ± 0.10 ^A^	2.31 ± 0.14 ^A^	0.81 ± 0.03 ^B^	6.79 ± 0.12 ^A^	31.3 ± 0.44 ^B^	23.2 ± 1.59 ^A^	45.6 ± 1.86

FA = Fatty Acid; SFA = Saturated Fatty Acid; MUFA = Monounsaturated Fatty Acid; PUFA = Polyunsaturated Fatty Acid; C1 = Yukon-R; C2 = DKC-2628; S1 = Big Fellow RR; S2 = Game Keeper RR; S3 = Kester’s Bob White Trailing Soybean; S1C1 = Big Fellow RR + Yukon-R; S2C1 = Game Keeper RR + Yukon-R; S3C1 = Kester’s Bob White Trailing Soybean + Yukon-R; S1C2 = Big Fellow RR + DKC-2628; S2C2 = Game Keeper RR + DKC-2628; S3C2 = Kester’s Bob White Trailing Soybean + DKC-2628. Mean values in each column followed by the same letter are not significantly different at (LSD, 0.05). Mono C = Monocropping Corn; Mono S = Monocropping Soybean; Mono-(US) = Monocropping Upright Soybean; Mono-(VS) = Monocropping Vine Soybean; Inter-(US) = Upright Soybean Intercropped with Corn; Inter-(VS) = Vine Soybean Intercropped with Corn.

**Table 9:** Pearson correlation coefficients between fodder fatty acids and quality.

	C16:0	C16:1n7	C18:0	C18:1n9	C18:2n-6	C18:3n-3	TFA
CP	0.62 ***	0.64 ***	0.86 ***	−0.36 **	−0.35 **	0.86 ***	0.31 *
AP	0.65 ***	0.64 ***	0.83 ***	−0.31 *	−0.37 **	0.82 ***	0.31 *
SP	−0.15 ^ns^	−0.46 ***	−0.19 ^ns^	0.50 ***	0.05 ^ns^	−0.20 ^ns^	0.13 ^ns^
ADF	−0.28 *	0.49 ***	0.19 ^ns^	0.09 ^ns^	−0.55 ***	−0.14 ^ns^	−0.55 ***
NDF	−0.62 ***	−0.38 **	−0.71 ***	0.53 ***	0.07 ^ns^	−0.86 ***	−0.41 ***
WSC	−0.40 ***	−0.54 ***	−0.47 ***	0.44 ***	0.14 ^ns^	−0.58 ***	−0.17 ^ns^
SS	−0.09 ^ns^	−0.45 ***	−0.20 ^ns^	0.49 ***	0.08 ^ns^	−0.18 ^ns^	0.18 ^ns^
Ash	0.10 ^ns^	0.89 ***	0.52 ***	0.07 ^ns^	−0.81 ***	0.26 *	−0.37 **
TDN	0.20 ^ns^	−0.68 ***	−0.34 **	−0.12 ^ns^	0.70 ***	−0.03 ^ns^	0.52 ***
NEL	0.40 ***	−0.43 ***	−0.02 ^ns^	−0.37 **	0.61 ***	0.31 *	0.60 ***
NEM	0.25 *	−0.62 ***	−0.26 *	−0.19 ^ns^	0.69 ***	0.06 ^ns^	0.54 ***
NEG	0.24 *	−0.62 ***	−0.26 *	−0.19 ^ns^	0.69 ***	0.07 ^ns^	0.54 ***
DDM	0.28 *	−0.49 ***	−0.19 ^ns^	−0.10 ^ns^	0.55 ***	0.14 ^ns^	0.55 ***
DMI	0.64 ***	0.44 ***	0.77 ***	−0.54 ***	−0.11 ^ns^	0.90 ***	0.41 ***
RFV	0.65 ***	0.26 *	0.65 ***	-0.51 ***	0.05 ^ns^	0.86 ***	0.53 ***

ns = non-significant; * correlation is significant (*p* ≤ 0.05); ** (*p* ≤ 0.01); *** (*p* ≤ 0.001) (n = 66 for all parameters). Crude protein = CP; Available Protein = AP; Soluble Protein = SP; Acid Detergent Fiber = ADF; Neutral Detergent Fiber = NDF; Water Soluble Carbohydrates = WSC; Simple Sugars = SS; Total Digestible Nutrients = TDN; Net Energy for Lactation = NEL; Net Energy for Maintenance = NEM; Net Energy of Gain = NEG; Digestible Dry Matter = DDM; Dry Matter Intake = DMI; Relative Feed Value = RFV.

## References

[1] Eskandari H., Ghanbari A. (2009). Intercropping of maize (*Zea mays*) and cowpea (*Vigna sinensis*) as whole-crop forage: Effect of different planting pattern on total dry matter production and maize forage quality. Not. Bot. Horti Agrobot..

[2] Eslamizadeh A., Kashani A., Ata S., Siyadat A., Modhej A., Lak S. (2015). Study of soybean forage at different planting dates intercropped with corn. WALIA J..

[3] Geren H., Avcioglu R., Soya H., Kir B. (2008). Intercropping of corn with cowpea and bean: Biomass yield and silage quality. Afr. J. Biotechnol..

[4] Ananthi T., Amanullah M.M., Al-Tawaha A.R.M.S. (2017). A review on maize-legume intercropping for enhancing the productivity and soil fertility for sustainable agriculture in India. Adv. Environ. Biol..

[5] Nadeau E., Rustas B.O., Arnesson A., Swensson C. Maize silage quality on Swedish dairy and beef farms. Proceedings of the 14th International Symposium Forage Conservation.

[6] Masoero F., Rossi F., Pulimeno A.M. (2006). Chemical composition and in vitro digestibility of stalks, leaves and cobs of four corn hybrids at different phenological stages. Ital. J. Anim. Sci..

[7] Armstrong K.L., Albrecht K.A., Lauer J.G., Riday H. (2008). Intercropping corn with lablab bean, velvet bean, and scarlet runner bean for forage. Crop Sci..

[8] Filya I., Sucu E., Karabulut A. (2006). The effect of Lactobacillus buchneri on the fermentation, aerobic stability and ruminal degradability of maize silage. J. Appl. Microbiol..

[9] Tau M.S. (2005). Grazing Management in the Communal Rangelands of the Upper Thukela, KwaZulu-Natal. Master’s Thesis.

[10] Jayanegara A., Dewi S.P., Laylli N., Laconi E.B., Nahrowi N., Ridla M. (2016). Determination of cell wall protein from selected feedstuffs and its relationship with ruminal protein digestibility in vitro. Media Peternak..

[11] Blount A.R.S., Wright D.L., Sprenkel R.K., Hewitt T.D., Myer R.O. (2009). Forage Soybeans for Grazing, Hay and Silage.

[12] Camacho Barrón M., González De Mejía E. (1998). Comparative study of enzymes related to proline metabolism in tepary bean (*Phaseolus acutifolius*) and common bean (*Phaseolus vulgaris*) under drought and irrigated conditions, and various urea concentrations. Plant Foods Hum. Nutr..

[13] Paulson J., Jung H., Raeth-Knight M., Linn J. (2008). Grass vs Legume Forages for Dairy Cattle.

[14] Baghdadi A., Halim R.A., Ghasemzadeh A., Ebrahimi M., Othman R., Yusof M.M. (2016). Effect of intercropping of corn and soybean on dry matter yield and nutritive value of forage corn. Legum. Res..

[15] Agarwal D.K., Billore S.D., Sharma A.N., Dupare B.U., Srivastava S.K. (2013). Soybean: Introduction, improvement, and utilization in India-problems and prospects. Agric. Res..

[16] Lithourgidis A.S., Vasilakoglou I.B., Dhima K.V., Dordas C.A., Yiakoulaki M.D. (2006). Forage yield and quality of common vetch mixtures with oat and triticale in two seeding ratios. Field Crop. Res..

[17] Ross S.M., King J.R., O’Donovan J.T., Spaner D. (2004). Intercropping berseem clover with barley and oat cultivars for forage. Agron. J..

[18] Javanmard A., Nasab A.D.M., Javanshir A., Moghaddam M., Janmohammadi H. (2009). Forage yield and quality in intercropping of maize with different legumes as double-cropped. J. Food Agric. Environ..

[19] Zhou X., Yu G., Wu F. (2011). Effects of intercropping cucumber with onion or garlic on soil enzyme activities, microbial communities and cucumber yield. Eur. J. Soil Biol..

[20] Brooker R.W., Jones H.G., Paterson E., Watson C., Brooker R.W., Bennett A.E., Cong W., Daniell T.J., George T.S., Hallett P.D. (2015). Improving intercropping: A synthesis of research in agronomy, plant physiology and ecology. New Phytol..

[21] Li L., Zhang L., Zhang F. (2013). Crop mixtures and the mechanisms of overyielding. Encycl. Biodivers..

[22] Jahanzad E., Sadeghpour A., Hosseini M.B., Barker A.V., Hashemi M., Zandvakili O.R. (2014). Silage yield and nutritive value of millet–soybean intercrops as influenced by nitrogen application. Agron. J..

[23] Htet M.N.S., Ya-qin P., Ya-dong X., Soomro R.N., Jiang-bo H. (2016). Effect of intercropping maize (*Zea mays* L.) with soybean (*Glycine max* L.) on green forage yield, and quality evaluation. IOSR J. Agric. Vet. Sci..

[24] Htet M.N.S., Soomro R.N., Jiang-bo H. (2017). Effects of different planting pattern of maize (*Zea mays* L.) and soybean (*Glycine max* (L.) Merrill) intercropping in resource consumption on fodder yield, and silage quality. Am. J. Plant Sci..

[25] Serbester U., Akkaya M.R., Yucel C., Gorgulu M. (2015). Comparison of yield, nutritive value, and in vitro digestibility of monocrop and intercropped corn-soybean silages cut at two maturity stages. Ital. J. Anim. Sci..

[26] Reta Sánchez D.G., Espinosa Silva J.T., Palomo Gil A., Serrato Corona J.S., Cueto Wong J.A., Gaytán Mascorro A. (2010). Forage yield and quality of intercropped corn and soybean in narrow strips. Span. J. Agric. Res..

[27] Yucel C., Avcı M., Inal I., Yucel D. (2017). Yield and silage quality of soybean-maize intercrop under different mixing ratios and harvest stages. Int. J. Agron. Agric. Res..

[28] Sanborn P., Lamontagne L., Hendershot W. (2011). Podzolic soils of Canada: Genesis, distribution, and classification. Can. J. Soil Sci..

[29] Harris L., Hiller J. (2018). Newfoundland and Labrador—Climate. Britannica.com.

[30] Van Der Heijden M.G.A., Bardgett R.D., Van Straalen N.M. (2008). The unseen majority: Soil microbes as drivers of plant diversity and productivity in terrestrial ecosystems. Ecol. Lett..

[31] Richardson A.E., Barea J.M., McNeill A.M., Prigent-Combaret C. (2009). Acquisition of phosphorus and nitrogen in the rhizosphere and plant growth promotion by microorganisms. Plant Soil.

[32] Bonkowski M. (2004). Protozoa and plant growth: The microbial loop in soil revisited. New Phytol..

[33] Elgersma A., Tamminga S., Ellen G. (2006). Modifying milk composition through forage. Anim. Feed Sci. Technol..

[34] Dewhurst R.J., Shingfield K.J., Lee M.A., Scollan N.D. (2006). Increasing the concentrations of beneficial polyunsaturated fatty acids in milk produced by dairy cows in high-forage systems. Anim. Feed Sci. Technol..

[35] Scollan N.D., Choi N.-J., Kurt E., Fisher A.V., Enser M., Wood J.D. (2001). Manipulating the fatty acid composition of muscle and adipose tissue in beef cattle. Br. J. Nutr..

[36] Pariza M.W. (2004). Perspective on the safety and effectiveness of conjugated linoleic acid. Am. J. Clin. Nutr..

[37] Ali W., Nadeem M., Ashiq W., Zaeem M., Thomas R., Kavanagh V., Cheema M. (2019). Forage yield and quality indices of silage-corn following organic and inorganic phosphorus amendments in podzol soil under boreal climate. Agronomy.

[38] Ali W., Nadeem M., Ashiq W., Zaeem M., Gilani S.S.M., Rajabi-Khamseh S., Pham T.H., Kavanagh V., Thomas R., Cheema M. (2019). The effects of organic and inorganic phosphorus amendments on the biochemical attributes and active microbial population of agriculture podzols following silage corn cultivation in boreal climate. Sci. Rep..

[39] Nadeem M., Pham T.H., Thomas R., Galagedara L., Kavanagh V., Zhu X., Ali W., Cheema M. (2019). Potential role of root membrane phosphatidic acid in superior agronomic performance of silage-corn cultivated in cool climate cropping systems. Physiol. Plant..

[40] Zaeem M., Nadeem M., Pham T.H., Ashiq W., Ali W., Gilani S.S.M., Elavarthi S., Kavanagh V., Cheema M., Galagedara L. (2019). The potential of corn-soybean intercropping to improve the soil health status and biomass production in cool climate boreal ecosystems. Sci. Rep..

[41] Weiss W.P., Conrad H.R., St. Pierre N.R. (1992). A theoretically-based model for predicting total digestible nutrient values of forages and concentrates. Anim. Feed Sci. Technol..

[42] Council N.R. (2001). Nutrient Requirements of Dairy Cattle.

[43] Van Soest P.J. (1982). Nutritional Ecology of the Ruminant.

[44] Folch J., Lees M., Stanley G. (1957). A simple method for the isolation and purification of total lipides from animal tissues. J. Biol. Chem..

[45] Fried B., Sherma J., Fried B. (2003). Handbook of Thin-Layer Chromatography.

[46] Liu J.H., Zeng Z.H., Jiao L.X., Hu Y.G., Wang Y., Li H. (2006). Intercropping of different silage maize cultivars and alfalfa. Acta Agron. Sin..

[47] Lithourgidis A.S., Dhima K.V., Vasilakoglou I.B., Dordas C.A., Yiakoulaki M.D. (2007). Sustainable production of barley and wheat by intercropping common vetch. Agron. Sustain. Dev..

[48] Abdulraheem M.I., Ojeniyi S.O., Charles E.F. (2012). Effect of different planting pattern on total dry matter production and maize forage quality in maize (*Zea mays*) and cowpea (*Vigna sinensis*) intercropped as whole-crop forage. IOSR J. Agric. Vet. Sci..

[49] Anil L., Park J., Phipps R.H. (2000). The potential of forage-maize intercrops in ruminant nutrition. Anim. Feed Sci. Technol..

[50] Dahmardeh M., Ghanbari A., Syasar B., Ramroudi M., Zhu Y., Bai C.S., Guo X.S., Xue Y.L., Ataku K. (2009). Effect of intercropping maize (*Zea mays* L.) with cow pea (*Vigna unguiculata* L.) on green forage yield and quality evaluation. Asian J. Plant Sci..

[51] Strydhorst S.M., King J.R., Lopetinsky K.J., Harker K.N. (2008). Forage potential of intercropping barley with faba bean, lupin, or field pea. Agron. J..

[52] Bingol N.T., Karsli M.A., Yilmaz I.H., Bolat D. (2007). The effects of planting time and combination on the nutrient composition and digestible dry matter yield of four mixtures of vetch varieties intercropped with barley. Turkish J. Vet. Anim. Sci..

[53] Contreras-Govea F.E., Albrecht K.A., Muck R.E. (2006). Spring yield and silage characteristics of kura clover, winter wheat, and in mixtures. Agron. J..

[54] Aasen A., Baron V.S., Clayton G.W., Dick A.C., McCartney D.H. (2004). Swath grazing potential of spring cereals, field pea and mixtures with other species. Can. J. Plant Sci..

[55] Lauriault L.M., Kirksey R.E. (2004). Yield and nutritive value of irrigated winter cereal forage grass-legume intercrops in the Southern High Plains, USA. Agron. J..

[56] Sleugh B., Moore K.J., George J.R., Brummer E.C. (2000). Binary legume-grass mixture improve forage yield, quality, and seasonal distribution. Agron. J..

[57] Costa P.M., Villela S.D.J., de Paula Leonel F., do Carmo Araújo S.A., Araújo K.G., Ruas J.R.M., Coelho F.S., Andrade V.R. (2012). Intercropping of corn, brachiaria grass and leguminous plants: Productivity, quality and composition of silages. Rev. Bras. Zootec..

[58] Gill K.S., Omokanye A.T. (2018). Potential of spring barley, oat and triticale intercrops with field peas for forage production, nutrition quality and beef cattle diet. J. Agric. Sci..

[59] Mugweni B.Z., Titterton M., Maasdorp B.V., Gandiya A.F. Effect of mixed cereal-legume silages on milk production from lactating holstein dairy cows (R7010). Proceedings of the 3rd Workshop Livestock Production Programme Projects.

[60] Htet M.N.S., Soomro R.N., Jiang-bo H. (2016). Intercropping of maize and climbing bean: Fodder yield, quality and nutrient composition of silages. Int. J. Agron. Agric. Res..

[61] Sadeghpour A., Jahanzad E., Esmaeili A., Hosseini M.B., Hashemi M. (2013). Forage yield, quality and economic benefit of intercropped barley and annual medic in semi-arid conditions: Additive series. Field Crop. Res..

[62] Salama H.S.A., Zeid M.M.K. (2016). Hay quality evaluation of summer grass and legume forage monocultures and mixtures grown under irrigated conditions. Aust. J. Crop Sci..

[63] Yurchak T., Orkine E. (2004). Beef ration rules of thumb agdex (420/52-4). Alberta Agric. Food Rural Dev. Publ..

[64] Rostamza M., Chaichi M.R., Jahansouz M.R., Alimadadi A. (2011). Forage quality, water use and nitrogen utilization efficiencies of pearl millet (*Pennisetum americanum* L.) grown under different soil moisture and nitrogen levels. Agric. Water Manag..

[65] Sadeghpour A., Jahanzad E., Lithourgidis A., Hashemi M. (2014). Forage yield & quality of barley-annual medic intercrops in semi-arid environments. Int. J. Plant Prod..

[66] Stoltz E., Nadeau E., Wallenhammar A.C. (2013). Intercropping maize and faba bean for silage under Swedish climate conditions. Agric. Res..

[67] Ullah M.A. (2010). Forage Production in Panicum Grass-Legumes Intercropping by Combining Geometrical Configuration, Inoculation and Fertilizer under Rainfed Conditions. Ph.D. Thesis.

[68] Caballero R., Goicoechea E.L., Hernaiz P.J. (1995). Forage yields and quality of common vetch and oat sown at varying seeding ratios and seeding rates of vetch. Field Crop. Res..

[69] Schroeder J.W. (1996). Quality Forage for Maximum Production and Return. North Dakota State Univ. Coop. Ext. Serv. Pub..

[70] Carr P.M., Horsley R.D., Poland W.W. (2004). Barley, oat, and cereal-pea mixtures as dryland forages in the northern Great Plains. Agron. J..

[71] National Research Council (2000). Nutrient Requirements of Beef Cattle.

[72] Iba K. (2002). Acclimative response to temperature stress in higher plants: Approaches of gene engineering for temperature tolerance. Annu. Rev. Plant Biol..

[73] Routaboul J.M., Skidmore C., Wallis J.G., Browse J. (2012). Arabidopsis mutants reveal that short-and long-term thermotolerance have different requirements for trienoic fatty acids. J. Exp. Bot..

[74] Falcone D.L., Ogas J.P., Somerville C.R. (2004). Regulation of membrane fatty acid composition by temperature in mutants of Arabidopsis with alterations in membrane lipid composition. BMC Plant Biol..

[75] Elgersma A. (2015). Grazing increases the unsaturated fatty acid concentration of milk from grass-fed cows: A review of the contributing factors, challenges and future perspectives. Eur. J. Lipid Sci. Technol..

[76] Khan N.A., Cone J.W., Fievez V., Hendriks W.H. (2012). Causes of variation in fatty acid content and composition in grass and maize silages. Anim. Feed Sci. Technol..

[77] Hatfield R.D., Jung H.J.G., Broderick G., Jenkins T.C. (2007). Nutritional chemistry of forages. Forages: The Science of Grassland Agriculture.

[78] Kalac P., Samkova E. (2010). The effects of feeding various forages on fatty acid composition of bovine milk fat: A review. Czech J. Anim. Sci..

[79] Khan N.A., Yu P., Ali M., Cone J.W., Hendriks W.H. (2015). Nutritive value of maize silage in relation to dairy cow performance and milk quality. J. Sci. Food Agric..

[80] Kliem K.E., Morgan R., Humphries D.J., Shingfield K.J., Givens D.I. (2008). Effect of replacing grass silage with maize silage in the diet on bovine milk fatty acid composition. Animal.

[81] Mach N., Zom R.L.G., Widjaja H.C.A., van Wikselaar P.G., Weurding R.E., Goselink R.M.A., van Baal J., Smits M.A., van Vuuren A.M. (2013). Dietary effects of linseed on fatty acid composition of milk and on liver, adipose and mammary gland metabolism of periparturient dairy cows. J. Anim. Physiol. Anim. Nutr..

[82] Enser M., Hallett K.G., Hewett B., Fursey G.A.J., Wood J.D., Harrington G. (1998). Fatty acid content and composition of UK beef and lamb muscle in relation to production system and implications for human nutrition. Meat Sci..

[83] Harfoot C.G., Hazlewood G.P., Hobson P.N., Stewart C.S. (1997). Lipid Metabolism in the Rumen. The Rumen Microbial Ecosystem.

[84] Lopes C.N., Scarpa A.B., Cappellozza B.I., Cooke R.F., Vasconcelos J.L.M. (2009). Effects of rumen-protected polyunsaturated fatty acid supplementation on reproductive performance of Bos indicus beef cows. J. Anim. Sci..

[85] De Veth M.J., Bauman D.E., Koch W., Mann G.E., Pfeiffer A.M., Butler W.R. (2009). Efficacy of conjugated linoleic acid for improving reproduction: A multi-study analysis in early-lactation dairy cows. J. Dairy Sci..

[86] Belury M.A. (2002). Inhibition of carcinogenesis by conjugated linoleic acid: Potential mechanisms of action. J. Nutr..

[87] Rochfort S., Parker A.J., Dunshea F.R. (2008). Plant bioactives for ruminant health and productivity. Phytochemistry.

[88] Khan N.A., Farooq M.W., Ali M., Suleman M., Ahmad N., Sulaiman S.M., Hendriks W.H. (2015). Effect of species and harvest maturity on the fatty acids profile of tropical forages. J. Anim. Plant Sci..

